# Mineotaur: a tool for high-content microscopy screen sharing and visual analytics

**DOI:** 10.1186/s13059-015-0836-5

**Published:** 2015-12-17

**Authors:** Bálint Antal, Anatole Chessel, Rafael E. Carazo Salas

**Affiliations:** Genetics Department, University of Cambridge, Downing Street, Cambridge, CB2 3EH UK

## Abstract

High-throughput/high-content microscopy-based screens are powerful tools for functional genomics, yielding intracellular information down to the level of single-cells for thousands of genotypic conditions. However, accessing their data requires specialized knowledge and most often that data is no longer analyzed after initial publication. We describe Mineotaur (http://www.mineotaur.org), a open-source, downloadable web application that allows easy online sharing and interactive visualisation of large screen datasets, facilitating their dissemination and further analysis, and enhancing their impact.

**Electronic supplementary material:**

The online version of this article (doi:10.1186/s13059-015-0836-5) contains supplementary material, which is available to authorized users.

## Main text

Despite groundbreaking discoveries in genomics, the genomes of most organisms remain black boxes with the function of the majority of genes and gene products still unknown. High-throughput/high-content microscopy-based screening (HT/HCS) provides an increasingly powerful tool to discover and functionally annotate genes and biological pathways, and in less than a decade has led to several important discoveries, like the systematic genome-wide identification of genes important for mitosis, endocytosis, the cytoskeleton, and other fundamental processes (Chia et al. [1], Cotta-Ramusino et al. [2], Collinet, C. et al. [3], Neumann et al. [4] Graml et al. [5]).

A current limitation of HT/HCS projects is that even after they are finalised, just accessing and visualising their output requires specialist expertise in image and data analysis, limiting their use and accessibility. This, along with the lack of a standardized way to manage and share the biological big data generated by HT/HCS screens with the wider community, limit the community’s capacity to fully exploit the rich, quantitative functional genomic information contained in those projects and thus the return on the investment made in them (Earnshaw [6]).

Here we introduce Mineotaur, a web-based interactive visual analytics tool we developed to provide an efficient way to share the large (>10^6^ points) amounts of image-derived feature data acquired by HT/HCS, and to allow the scientific community complete access to easily visualize and inspect HT/HCS data, linking with images when available (detailed documentation of Mineotaur can be accessed at http://docs.mineotaur.org).

While there are open source [7] and commercial software (http://www.tableau.com/, http://www.moleculardevices.com/systems/high-content-imaging/acuityxpress-high-content-informatics-software, http://spotfire.tibco.com/) which allow visual analysis of HT/HCS data, Mineotaur is unique in that it lets scientists publish their screens in a standard way together with a free pre-packaged visual analytical toolkit. Once an instance has been set up the aim is for end users, for example biologists without deep computational knowledge, to be able to access data with a minimal investment of time and effort.

**Fig. 1:**
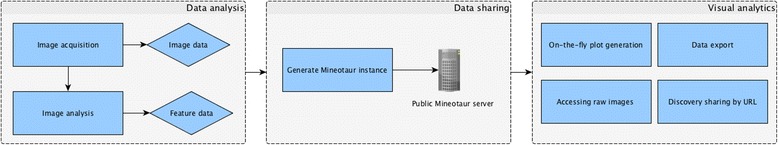
Pipeline to share high-throughput/high-content microscopy screens. The acquired images and feature data extracted from them can be used to generate a Mineotaur instance, allowing research groups to share their data in a standard way. The public Mineotaur servers let the interested members of the scientific community to gain an understanding and confidence of the data by accessing features and images in a few clicks, generating plots on-the-fly and provides a toolkit for researchers discover new hypotheses using the data and export and share supporting findings

The pipeline for data sharing and visual analytics using Mineotaur can be seen in Fig. 1. Mineotaur is based on a property graph model (Robinson et al. [8]) to handle HT/HCS screens, which is implemented using Java (http://java.oracle.com), and a graph database, Neo4j (http://www.neo4j.com) (see the Additional file 1: Figure S1, Figure S2, Figure 3 and the Online Methods for details of the data model and implementation). Neo4j is a database system built upon the concept of property graphs, which models all objects of interest (e.g. genotypes/cell lines, genes, images, cells, etc.) as nodes in a graph, and edges in the graph represent a connection between them (e.g. an edge between ‘cell’ nodes and an ‘image’ node signify ‘cells extracted from that image’). Each node also stores an arbitrary list of property values (e.g. for each cell, the value of each of the quantitative feature measurements extracted from it by the computational pipeline) and labels (e.g. a gene identified as a ‘hit’ for a given process in a HT/HCS screen can be annotated as implicated in that process), providing flexibility to different data sources and to updates/changes in the data, which is almost impossible in standard relational data models. For convenience, we provide a standard way to generate a graph model and a Mineotaur instance for any HT/HCS screen simply from a CSV (comma separated values) file. An example data model for the [5] screen can be seen in Additional file 1: Figure S1, while Additional file 1: Figure S4 and Additional file 1: Figure S5 shows how example input files for the Mineotaur instance generation need to be formulated.

**Fig. 2:**
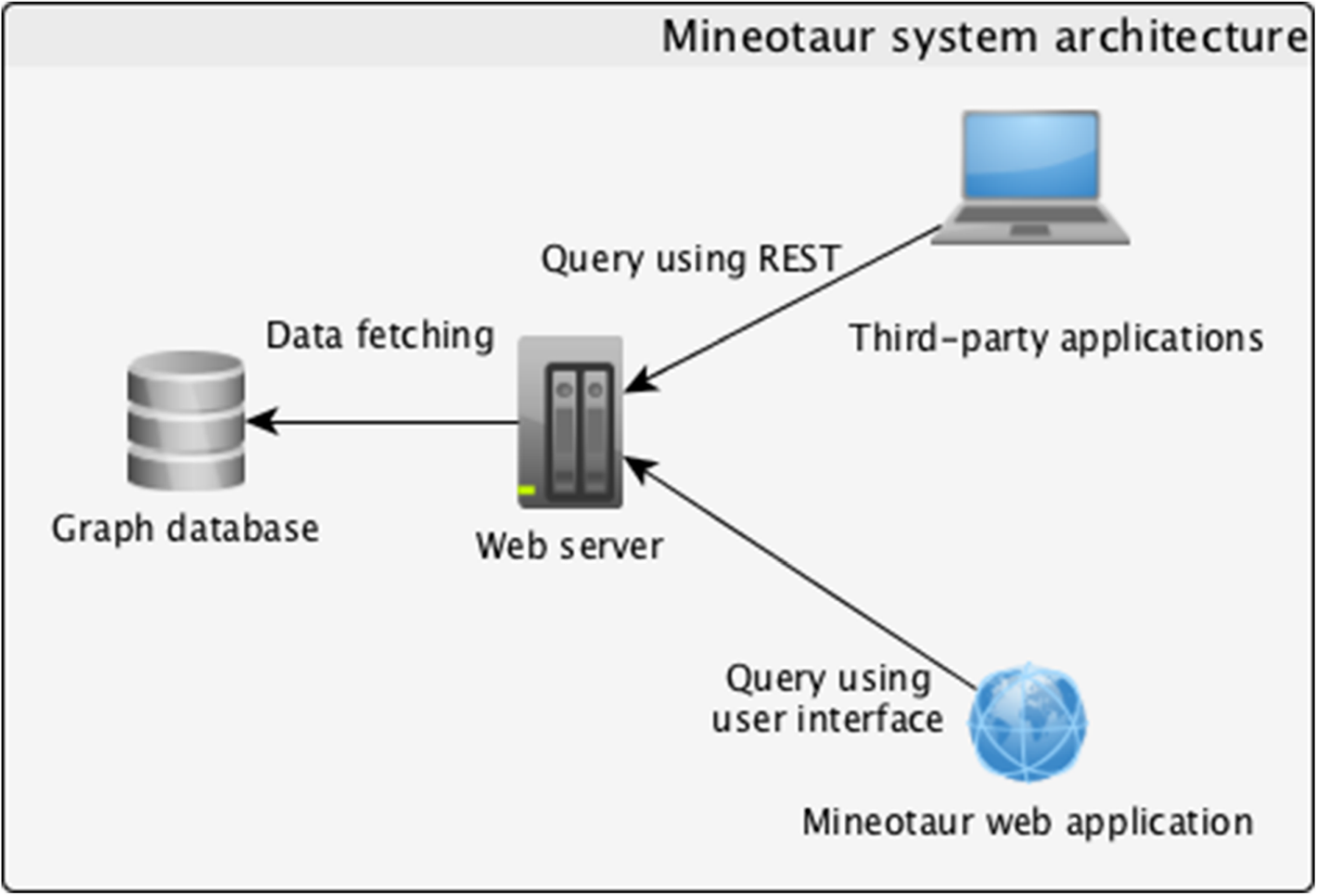
Architecture of the Mineotaur system. The Mineotaur web server can be accessed from both a web interface and programatically using REST. The web server handles the interaction with the graph database containing the HT/HCS data

**Fig. 3:**
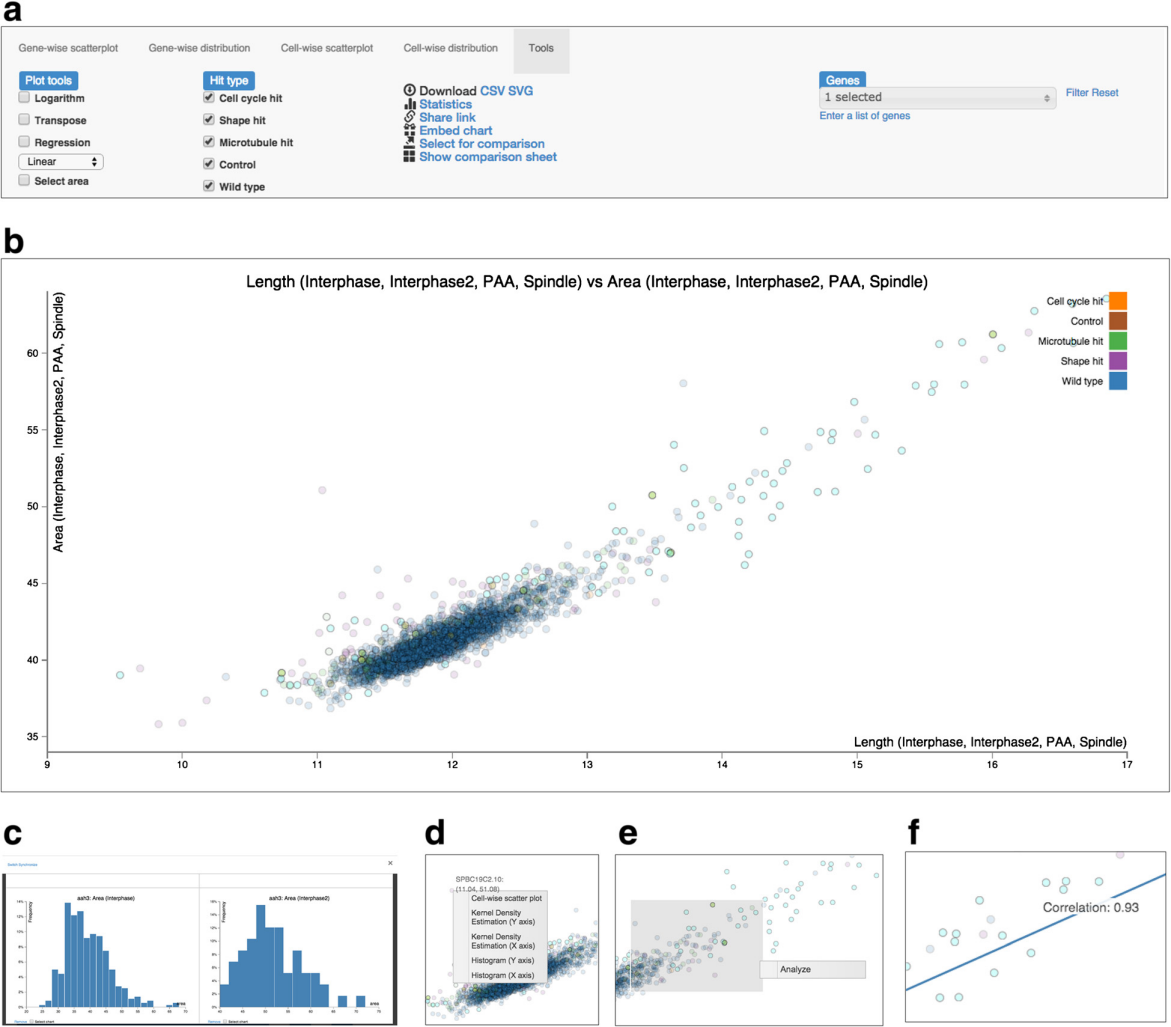
Unique distinguishing features of Mineotaur. **a** The web interface allows on-the-fly analysis of the data. **b** Queries are visualized as interactive plots. **c** Analytics tools like regression lines can be used to generate new hypotheses about the data. **d** Subsets of data can be selected for in depth analysis. **e** From a gene-level plot, cell-level information can be reached from a context menu in one click. **f** Multiple plots can be compared to one another using the plot comparison tool

The data stored in Mineotaur can be accessed by both an interactive web-based Graphical User Interface (GUI), which enables intuitive ad-hoc querying and plotting of the descriptive information extracted from the HT/HCS microscopy screens in real-time, and programmatically, using a REST interface (see Fig. 2 for the architecture of Mineotaur). Thus, the screen data can be exploited by both experimental and computational biologists. An example screen from Mineotaur’s web interface is shown in Fig. 3. As a design principle, we wanted to allow users to easily construct complex queries and traverse through to raw images or well-established genomic databases in a few clicks. Feature data can be accessed at multiple levels (e.g. genes and cells), if suitable data are available. The user can discover multidimensional associations between different descriptors by generating scatter plots with adjustable axes, or analyze the distribution of single features for certain conditions in the form of (multi)histograms and kernel density estimation plots. From scatter plots, the users can access the raw images if they are available online (for example using Omero [9]) with a single click on the data points. Furthermore, users can jump from gene-level scatter plots to extracting cell-level knowledge with a single click.

The queries can be transformed by filtering the data points at the different levels of the investigated conditions (e.g. gene or cells annotations). The queried data can be further analysed by allowing area selection within a plot, regression line fitting, plot transformation and plot comparison.

To ensure the reusability and reproducibility of the data analysis, Mineotaur users can generate a link allowing them to share the plots they generate (including any filters applied), as well as export their data to different formats such as vector graphical format SVG (to export plots) or a comma separated CSV text file containing the raw data values, for use with standard spreadsheet tools (for an explanation of how to export data see http://docs.mineotaur.org/en/latest/plot_tools.html#download). We also provide a way to enrich publications by allowing users to embed interactive charts from Mineotaur to web pages (to be used e.g. as an interactive figure in the HTML version of an online material in journals). The querying capabilities of Mineotaur can be seen in Additional file 2: video S1, while an example post-query can be seen in Additional file 3: video S2. The code is open-source and is accessible at https://github.com/antalbalint/Mineotaur/ under GPL license.

To demonstrate the capabilities and versatility of the tool, we used data from two published screens. First, a genomic multi-process HT/HCS screen recently published by our group [5], containing quantitative phenotypic annotations of hundreds of genes influencing cell shape, microtubules and cell cycle progression in fission yeast (*Schizosaccharomyces pombe*). The screen consists of images from tens of 96-well plates, with each well containing cell populations knocked out for a specific non-essential *S. pombe* gene (except for a few wells containing positive/negative controls). The data consists of 138 000 images, from which 1.7 million cells and 5.5 million microtubules were computationally identified and quantitated, leading to 131 features extracted from each cell. For details on the experimental pipeline and its connection to Mineotaur see Methods and Additional files 1, 2, 3, 4 and 5. Secondly, we also generated a Mineotaur instance from a subset of an HT/HCS screen investigating the signalling network controlling the Golgi apparatus in human cells [10], which contains 624 features for 1 580 242 cells, imaged from 353 different conditions. Additional file 4: video S3 shows the step-by-step reconstruction of a figure from [5], while the reconstructed figure can be seen in Additional file 1: Figure S6. A demonstration instance for the latter dataset can be accessed at http://demo.mineotaur.org/.

In summary, we have developed Mineotaur, a graph model based web application, allowing visual analytics and sharing of HT/HCS projects amongst the entire community, computational and non-computational alike. We believe the intuitive interface, versatility and scalability will greatly potentiate the return-on-investment of past and future projects in the field, catalyse deep biological advances and open the way to establishing community-wide data and interoperability standards.

## Materials & methods

### The graph model

#### Basic concepts

Property graph: a mathematical graph where each nodes and vertices can also hold tuples of data.

Objects of interest: any part of an experiment to be included in Mineotaur to be either directly queried or stored as metadata. Example: Strain, Gene, Cell, Experiment.

Grouping object: the main object of interest, the top object in the graph. Example: Gene, Strain.

Descriptive objects: the objects carrying the detailed data associated with the grouping object. Example: Cell. Please note that in cases where only one layer of experimental data is available, the grouping objects can be descriptive objects as well.

### Import data

The graph model for each Mineotaur instance can be generated by providing a CSV (comma separated value) file in the following format:

- First line: column headers. These will serve as the property names for their respective object types in the database.
- Second line: object names. These describe the names of the objects of interest to be stored in the database. The property in each column described in the first line will be associated with the object described here.
- Third line: property types. These describe the data types of the properties for each column. The possible values are:
  - TEXT: the column contains a text. Stored as a metadata.
  - NUMBER: the column contains a number, thus it will be become a queryable information if stored in a descriptive object.
  - ID: identifier of the object. Multiple ID properties can be set to an object.
  - URL: the URL of the resource to be linked to object.

Each line after the third provides a descriptive object instance.

For an example input file, see Additional file 1: Figure S4.

To provide annotation for the grouping objects, an additional input file containing the labels is needed. The first n column describe the IDs for the grouping object. All other columns provide a binary value for a label (1=the grouping object possesses the annotation, 0=otherwise). An example label input file can be seen in Additional file 1: Figure S5.

### Detailed architecture

Server side: The server side application is written in Java and based on the Spring framework. On the server side, the data handling and the business logic behind the application is separated by design. As a graph database, Neo4J is used in embedded mode. The server handles incoming HTTP requests for the data, translate it to the data model and fetches data from the graph database. The data is sent back either as a HTTP response or a JSON file.

Client side: The server can be accessed in two ways: by the user interface of the Mineotaur and by REST.

The user interface is based on HTML 5 (generated from Thymeleaf templates by the server), Bootstrap and Javascript. The user interface provides 1) an interface for ad-hoc querying the data 2) visualizes the response of the server 3) provides tools to analyse the data. When a query is submitted, an asynchronous call to the server is sent using AJAX. The server provides a response in JSON format, from which the appropriate plot is rendered using the D3 javascript library. The analytic toolkit of the user interface is built using the jQuery library and designed as a modular Model-View-Controller (MVC) application (see Additional file 1: Figure S3 for the architecture) using the require.js AMD (Anyschronous Module Definition) framework. The client side consists of the following Javascript modules:

- Controller: handling all events triggered in the user interface.
- Context: a data access object containing all relevant information for the current session
- UI: all functions to provide an interactive user interface
- Plot: functions required for generating the plots using the D3 framework.
- Utilities: common mathematical functions used throughout the application

## REST

The server side can be accessed programmatically from any programming language or framework capable of handling HTTP requests and responses and JSON (i.e. Java, Python, Matlab, Bash, etc.).

## Additional files

Additional file 1:
**Supplemental Figures S1–S6.** (DOCX 1246 kb) Additional file 2:
**Video S1.** Pre-query selection options of Mineotaur. (MOV 1868 kb) Additional file 3:
**Video S2.** Post-query selection options of Mineotaur: a) Plot transformations b) Filtering c) Connecting plots d) Connecting to external software and website. (MOV 13448 kb) Additional file 4:
**Video S3.** Reconstruction of Figure 7a from [5]. (MOV 1136 kb) Additional file 5:
**Mineotaur documentation for Release 1.0.1.** (PDF 1303 kb)
