# Supplementary material for: Mineotaur: a tool for high-content microscopy screen sharing and visual analytics
Source: Genome Biol. 2015 Dec 17;16:283. doi: 10.1186/s13059-015-0836-5 (PMC4699365; doi:10.1186/s13059-015-0836-5)
Supplement: Additional file 1: — Supplemental Figures S1–S6. (DOCX 1246 kb) [file 13059_2015_836_MOESM1_ESM.docx]

**Supplementary Figures**


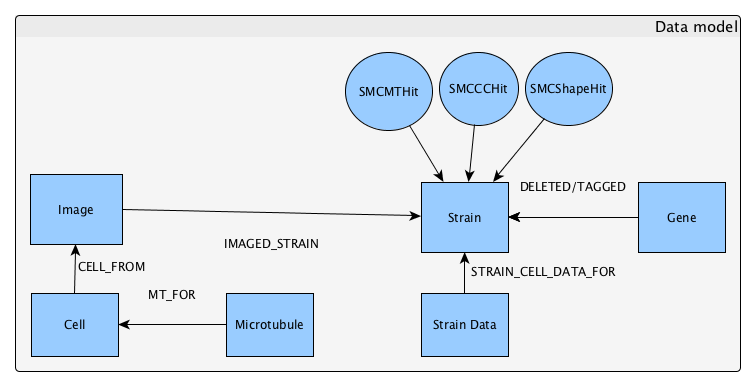


Figure S1: **Graph-based data model behind Mineotaur, as applied to the Graml et al study.** The concepts in the rectangles represent nodes, the circles represent labels and whereas the arrows between represent edges. The central concept of the model is the strain, which has an associated deleted or tagged Gene associated to it, serving as conditions in the study. The strains are connected to the image nodes, containing all relevant metadata from the images taken from the appropriate conditions, and also providing access to the cell and microtubule level features extracted from the images. The ‘Strain data’ nodes contain aggregated data to be speed up querying, while each strain node has a hit type assigned based on the paper.


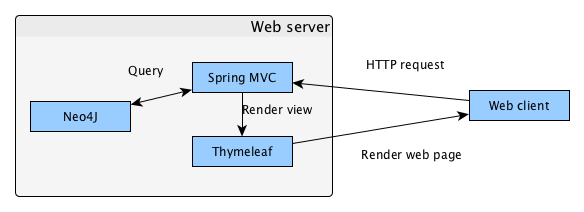


Figure S2: **Architecture of the server side.** The web server if based on the Spring Model-View-Controller (MVC), using Thymeleaf as a template engine. The data is stored in the Neo4j graph database. A web client can access the content by making an HTTP request to the server, which will query the appropriate data from the database and render a web page from a Thymeleaf template.


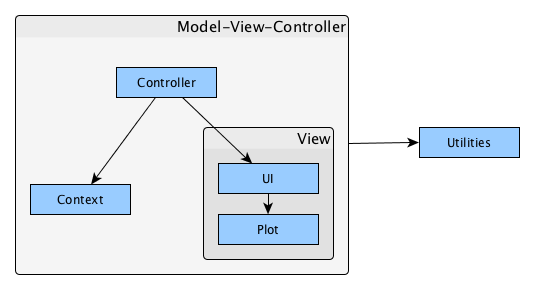


Figure S3: **Architecture of the client side.** On the client side, all interaction is done using a Javascript application. The application is modular, with different modules responsinble to handle events (Controller), carry data values (Context), manipulate web pages (UI), generate plots (Plot) and provide general functionalities (Utilities).


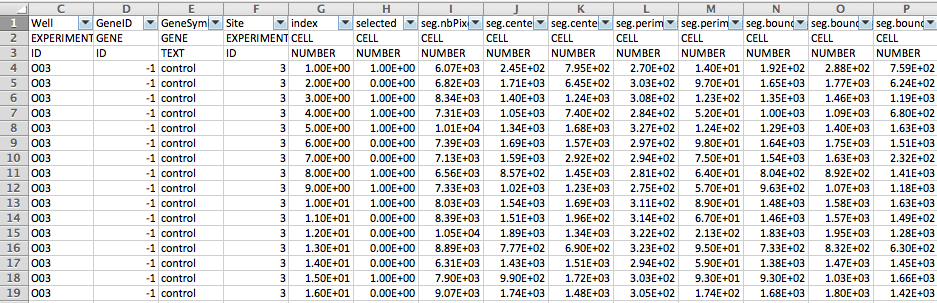


Figure S4: **Example input file fragment for the Chia et al. dataset.** The first row contains the header information, the next two lines describes the data contained in the appropriate columns and the rest of the file contain the data points. Input file creation is described in detail at <http://docs.mineotaur.org/en/latest/install.html>.


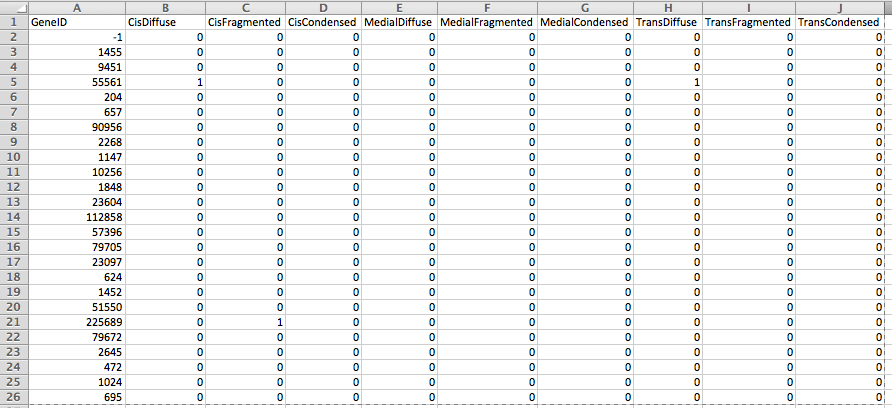


Figure S5: **Example annotation file for the Chia et al. dataset.** A value of 1 in a cell means the Gene represented by and identifier (row) has the corresponding label (column) and 0 otherwise.

**

**

Figure S6: Straightforward reconstruction of Figure 7a from (Graml et al., 2014) using Mineotaur. To watch an explanation of the reconstruction process of the plot see Supplementary Video 3.
