# Supplementary material for: Mineotaur: a tool for high-content microscopy screen sharing and visual analytics
Source: Genome Biol. 2015 Dec 17;16:283. doi: 10.1186/s13059-015-0836-5 (PMC4699365; doi:10.1186/s13059-015-0836-5)
Supplement: Additional file 5: — Mineotaur documentation for Release 1.0.1. (PDF 1303 kb) [file 13059_2015_836_MOESM5_ESM.pdf]

Mineotaur Documentation

Release 1.0.1

Balint Antal

August 25, 2015

1 Getting started 3
1.1 Motivation . . . 3
1.2 What is Mineotaur? . . . 3
1.3 About the documentation . . . 3

2 Installation 5
2.1 Requirements . . . 5
2.2 Generting a Mineotaur instance from text files . . . 5
2.3 Label file . . . 6
2.4 Options file . . . 7
2.5 Generation from command line . . . 7
2.6 Generation using the wizard . . . 8

3 Using the web interface 9
3.1 Layout . . . 9
3.2 Help . . . 10

4 Query tools 11
4.1 Variable selection . . . 11
4.2 Filtering by annotation . . . 13
4.3 Group selection . . . 13

5 Scatterplots 17
5.1 Using the scatterplot . . . 17
5.2 Plot tools . . . 19
5.3 Visual filtering of nodes . . . 23

6 Distribution plots 25
6.1 Histogram . . . 25
6.2 Multihistogram . . . 25
6.3 Kernel Density Estimation . . . 26

7 The Tools menu 27
7.1 Filtering by hit Type . . . 27
7.2 Other plot tools . . . 27

8 Developing Mineotaur 31
8.1 Software used to create Mineotaur . . . 31
8.2 Architecture of Mineotaur . . . 32

9 Features planned to be included in further releases of Mineotaur 35

10 Licence 37

11 Indices and tables 39

Mineotaur is a web application to share and visually analyse high-throughput/high-content microscopy screens developed at the Carazo Salas lab of the University of Cambridge, Department of Genetics.

The project website can be found at http://www.mineotaur.org. Please cite the following paper when using Mineotaur: B. Antal, A. Chessel, R. E. Carazo Salas: Mineotaur: interactive visual analytics for high-content microscopy screens, under revision.

Contents:

CHAPTER 1

Getting started

1.1 Motivation

Despite the ground-breaking discoveries in genomics, the genomes of most organisms remain black boxes with the function of the majority of genes and gene products still unknown. Moreover, many genes and proteins play roles in multiple biological processes. High-throughput/high-content microscopy-based screening (HT/HCS) provides an increasingly powerful tool to discover and functionally annotate genes and biological pathways, which already led to several important discoveries, like the systematic identification of genes important for mitosis, endocytosis, and other fundamental processes. Specialised large-scale image and data analysis methods are needed to produce phenotypic data, limiting such functional genomic annotation techniques to researchers of groups that possess that expertise. This means that the community at large is limited in their access to data and their ability to further mine it after publication, reducing the impact of the expensive HT/HC screens. Overall, while technical advances led to an explosion in the amount of data being acquired, suitable data handling, visualization and analysis techniques are still lagging behind.

1.2 What is Mineotaur?

Here we propose a novel data visualization tool called Mineotaur (http://www.mineotaur.org), which will allow the community to mine further the raw multidimensional feature data and knowledge from published HT/HC screens leading to a better exploitation of experimental results. The user interface allows the members of the community without any computational knowledge to extract meaningful information from the data. The web interface can be used for querying the data and the results are visualized as plots (e.g. scatter plot, histogram) in real-time. The tool is based on a novel data model allowing the visualization and analysis of extremely large amounts of data.

1.3 About the documentation

Installation describes how to generate a new Minetaur instance. To use an existing Mineotaur instance, see Using the web interface. Those who want to understand the technical aspects of Mineotaur better or would like to contribute to it, go to Developing Mineotaur.

CHAPTER 2

Installation

2.1 Requirements

Mineotaur requires Java 8 or higher, which can be download here: http://www.oracle.com/technetwork/java/javase/downloads/index-jsp-138363.html

If you want to build Mineotaur from source, you will also need Maven: https://maven.apache.org/

2.2 Generting a Mineotaur instance from text files

To generate a Mineotaur instance, you have to provide three input files: a data file containing all the measurements you want to include in Mineotaur, a label file containing the annotations assigned to the objects in Mineotaur and a file setting several options in Mineotaur. A sample for all input file can be downloaded here..

2.2.1 Data file

The input data file can ?SV (? Separated Values), where ? is an appropriate separator set in the options file (e.g. TSV - Tab Separated Values). Each line describes a set of measurements for a descriptive object, which is a unique obejct of interest in the experiment. Each descriptive object should be connected to a group object. Examples: descriptive object - cell, group object - gene. The file is consists of a header, an object and a type descriptor and the data lines.

	C	D	E	F	G	H	I	J	K	L	M	N	O	P
1	Well	GeneID	GeneSym	Site	index	selected	seg.nbPix	seg.cente	seg.cente	seg.perim	seg.perim	seg.bound	seg.bound	seg.bound
2	EXPERIMENT	GENE	GENE	EXPERIMENT	CELL	CELL	CELL	CELL	CELL	CELL	CELL	CELL	CELL	CELL
3	ID	ID	TEXT	ID	NUMBER	NUMBER	NUMBER	NUMBER	NUMBER	NUMBER	NUMBER	NUMBER	NUMBER	NUMBER
4	O03	-1	control	3	1.00E+00	1.00E+00	6.07E+03	2.45E+02	7.95E+02	2.70E+02	1.40E+01	1.92E+02	2.88E+02	7.59E+02
5	O03	-1	control	3	2.00E+00	0.00E+00	6.82E+03	1.71E+03	6.45E+02	3.03E+02	9.70E+01	1.65E+03	1.77E+03	6.24E+02
6	O03	-1	control	3	3.00E+00	1.00E+00	8.34E+03	1.40E+03	1.24E+03	3.08E+02	1.23E+02	1.35E+03	1.46E+03	1.19E+03
7	O03	-1	control	3	4.00E+00	1.00E+00	7.31E+03	1.05E+03	7.40E+02	2.84E+02	5.20E+01	1.00E+03	1.09E+03	6.80E+02
8	O03	-1	control	3	5.00E+00	1.00E+00	1.01E+04	1.34E+03	1.68E+03	3.27E+02	1.24E+02	1.29E+03	1.40E+03	1.63E+03
9	O03	-1	control	3	6.00E+00	0.00E+00	7.39E+03	1.69E+03	1.57E+03	2.97E+02	9.80E+01	1.64E+03	1.75E+03	1.51E+03
10	O03	-1	control	3	7.00E+00	0.00E+00	7.13E+03	1.59E+03	2.92E+02	2.94E+02	7.50E+01	1.54E+03	1.63E+03	2.32E+02
11	O03	-1	control	3	8.00E+00	1.00E+00	6.56E+03	8.57E+02	1.45E+03	2.81E+02	6.40E+01	8.04E+02	8.92E+02	1.41E+03
12	O03	-1	control	3	9.00E+00	1.00E+00	7.33E+03	1.02E+03	1.23E+03	2.75E+02	5.70E+01	9.63E+02	1.07E+03	1.18E+03
13	O03	-1	control	3	1.00E+01	1.00E+00	8.03E+03	1.54E+03	1.69E+03	3.11E+02	8.90E+01	1.48E+03	1.58E+03	1.63E+03
14	O03	-1	control	3	1.10E+01	0.00E+00	8.39E+03	1.51E+03	1.96E+02	3.14E+02	6.70E+01	1.46E+03	1.57E+03	1.49E+02
15	O03	-1	control	3	1.20E+01	0.00E+00	1.05E+04	1.89E+03	1.34E+03	3.22E+02	2.13E+02	1.83E+03	1.95E+03	1.28E+03
16	O03	-1	control	3	1.30E+01	0.00E+00	8.89E+03	7.77E+02	6.90E+02	3.23E+02	9.50E+01	7.33E+02	8.32E+02	6.30E+02
17	O03	-1	control	3	1.40E+01	0.00E+00	6.31E+03	1.43E+03	1.51E+03	2.94E+02	5.90E+01	1.38E+03	1.47E+03	1.45E+03
18	O03	-1	control	3	1.50E+01	1.00E+00	7.90E+03	9.90E+02	1.72E+03	3.03E+02	9.30E+01	9.30E+02	1.03E+03	1.66E+03
19	O03	-1	control	3	1.60E+01	0.00E+00	9.07E+03	1.74E+03	1.48E+03	3.05E+02	1.74E+02	1.68E+03	1.80E+03	1.42E+03

Header

The first line of the data file. The header describes the names of the properties to be stored in the Mineotaur. Each name must be unique for a given object type and should not contain non-alphanumerical characters.

Object descriptor

The second line of the data file. The object descriptor describes what kind of real-world object does the respective column belongs to. The object descriptors can be any string. However it is advised to give semantically relevant names to future usage. Examples: Gene, Cell, Experiment.

Type descriptor

The third line of the data file. The type descriptor describes the data type for each column. The following types are accepted: * ID: identifier for a given object. Can be multiple IDs for one object type. * NUMBER: numerical data. Each numerical column of the descriptive can be queried. * TEXT: non-numerical data.

Data lines

Each line after starting from the fourth should contain the actual measurements for a descriptive object and other meatadat connecting them to experimental conditions.

2.3 Label file

The label file contain the annotations for the group level objects. For example, what genes were picked up as hits in a study. The label file consists of a header line and multiple label lines.

	A	B	C	D	E	F	G	H	I	J
1	GeneID	CisDiffuse	CisFragmented	CisCondensed	MedialDiffuse	MedialFragmented	MedialCondensed	TransDiffuse	TransFragmented	TransCondensed
2	-1	0	0	0	0	0	0	0	0	0
3	1455	0	0	0	0	0	0	0	0	0
4	9451	0	0	0	0	0	0	0	0	0
5	55561	1	0	0	0	0	0	1	0	0
6	204	0	0	0	0	0	0	0	0	0
7	657	0	0	0	0	0	0	0	0	0
8	90956	0	0	0	0	0	0	0	0	0
9	2268	0	0	0	0	0	0	0	0	0
10	1147	0	0	0	0	0	0	0	0	0
11	10256	0	0	0	0	0	0	0	0	0
12	1848	0	0	0	0	0	0	0	0	0
13	23604	0	0	0	0	0	0	0	0	0
14	112858	0	0	0	0	0	0	0	0	0
15	57396	0	0	0	0	0	0	0	0	0
16	79705	0	0	0	0	0	0	0	0	0
17	23097	0	0	0	0	0	0	0	0	0
18	624	0	0	0	0	0	0	0	0	0
19	1452	0	0	0	0	0	0	0	0	0
20	51550	0	0	0	0	0	0	0	0	0
21	225689	0	1	0	0	0	0	0	0	0
22	79672	0	0	0	0	0	0	0	0	0
23	2645	0	0	0	0	0	0	0	0	0
24	472	0	0	0	0	0	0	0	0	0
25	1024	0	0	0	0	0	0	0	0	0
26	695	0	0	0	0	0	0	0	0	0

The first line of the label file. The first column contains the name of the group object ID property from the data file, while the rest of the columns contain the annotations.

Each line starting from the second contains a group object ID and a 1 for each annotation assigned to the group object or 0, otherwise.

2.4 Options file

The options describes metadata for the instance generation. All options are in the following format: option_name = option_value. The following options can be set:

- (REQUIRED) name: name of the instance
- group: name of the group object (same as described in the data file). Default: GENE
- groupName: group object ID (same as described in the data file). Default: geneID
- descriptive: name of the group object (same as described in the data file). Default: CELL
- total_memory: the amount of memory can be used by Neo4J. Default: 4G
- separator: character used to separate columns in the data and the label files. Default: \t
- overwrite: whether to overwrite the current instance with the same name. Default: true

2.5 Generation from command line

1. Download the latest jar file from http://www.mineotaur.org.
2. Create a property file, a data file and a label file (see documentation and example input data)
3. Start the data import with the following command:

   java -jar <path_to_jar file> -import mineotaur.input chia_sample.tsv chia_labels.tsv
4. After the database creation is completed you can start your Mineotaur instance with the following command:

java –jar <path_to_jar file> -start <instance_name>

5. You can start querying at http://127.0.0.1:8080 in your browser.

2.6 Generation using the wizard

1. Download the latest jar file from http://www.mineotaur.org.
2. Create a property file, a data file and a label file (see documentation and example input data)
3. Start the data import with the following command:

   java -jar <path_to_jar file> -wizard

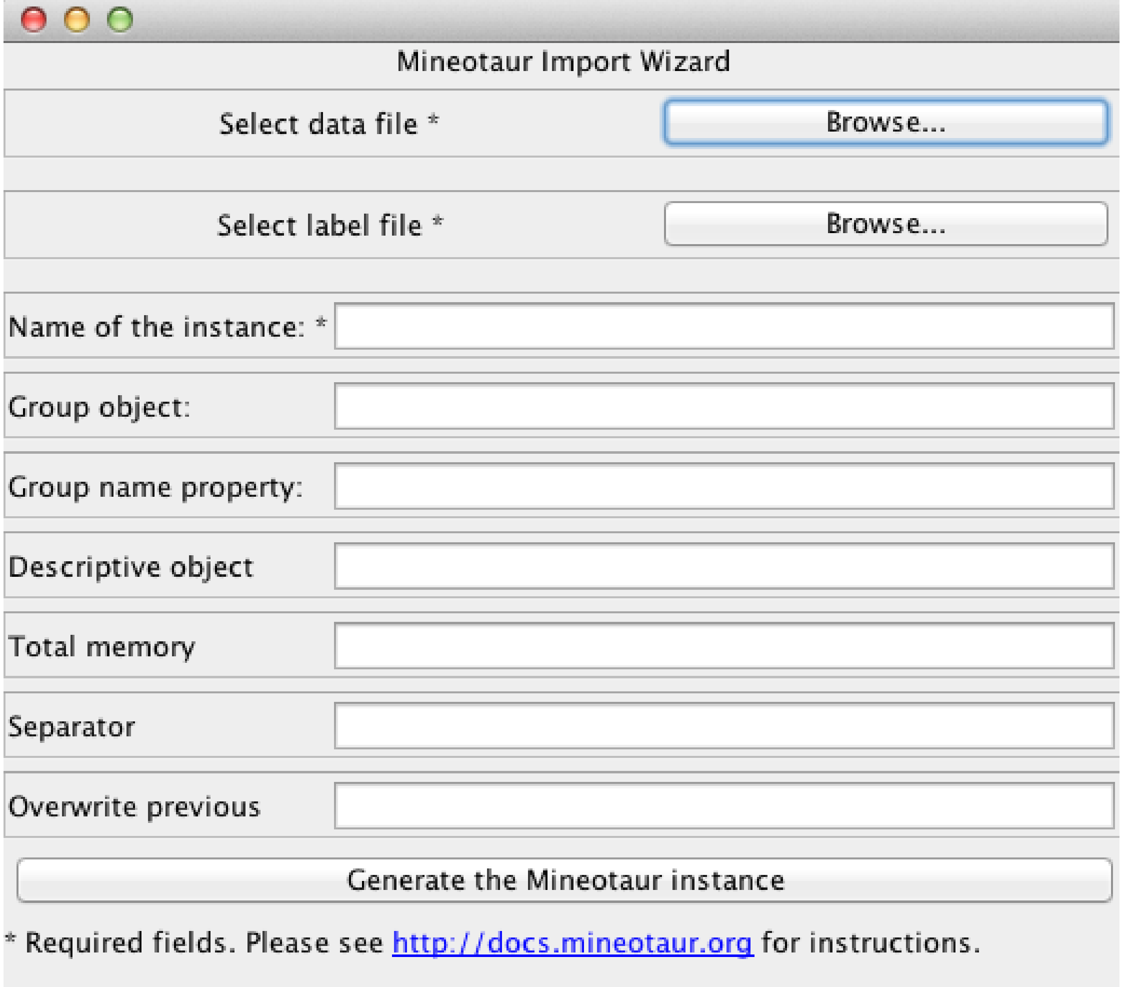

4. After the database creation is completed you can start your Mineotaur instance with the following command:

   java –jar <path_to_jar file> -start <instance_name>

5. You can start querying at http://127.0.0.1:8080 in your browser.

Using the web interface

3.1 Layout

Each Mineotaur instance use the same web interface layout.

3.1.1 Menu

The top element is the menu, showing the different querying tools which can be selected.

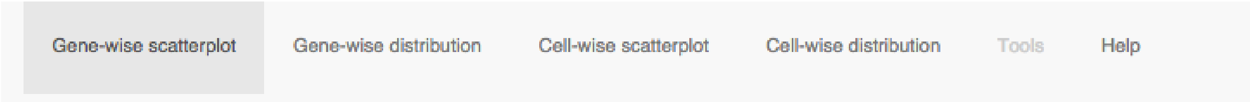

By clicking on any of the elements, the respective query panel will be activated and shown.

3.1.2 Query panel

In each query panel, a variety of different option can be set to customize the query.

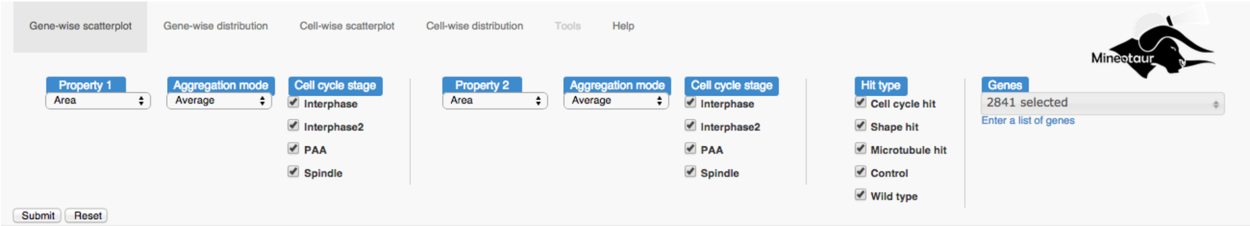

For more details, please go to the Query tools, Scatterplots and the Distribution plots pages.

3.1.3 Plot area

The plot showing the requested data will be shown below the query panel.

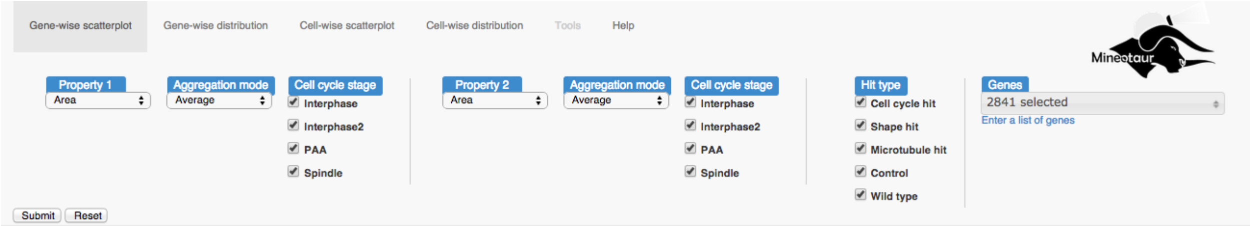

After the query, the Tools menu is activated, which allows different actions regarding the plot and the queried data.

For more details, please go to the The Tools menu, Scatterplots and the Distribution plots pages.

3.2 Help

(Optional) If provided with the Mineotaur instance, clicking on the Help link show information on the elements shown on the current page.

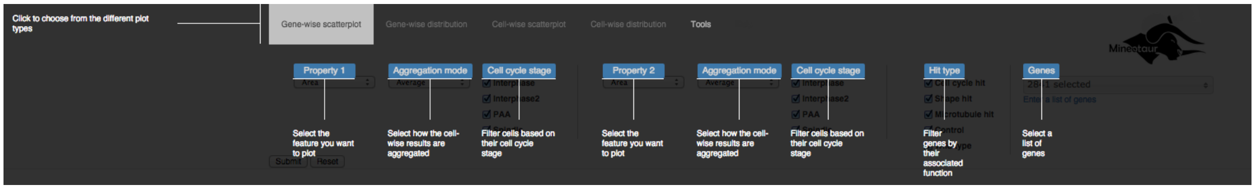

Query tools

4.1 Variable selection

First, the two variables (properties) to be shown needs to be selected.

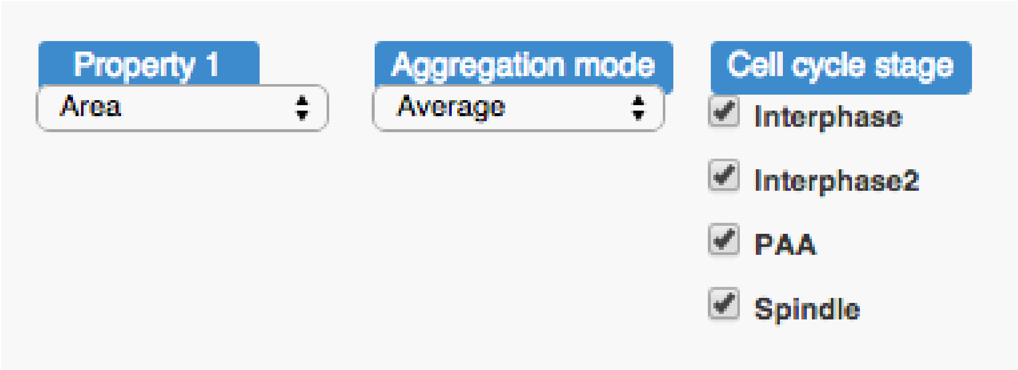

Clicking on the selection panel shows the available variables.

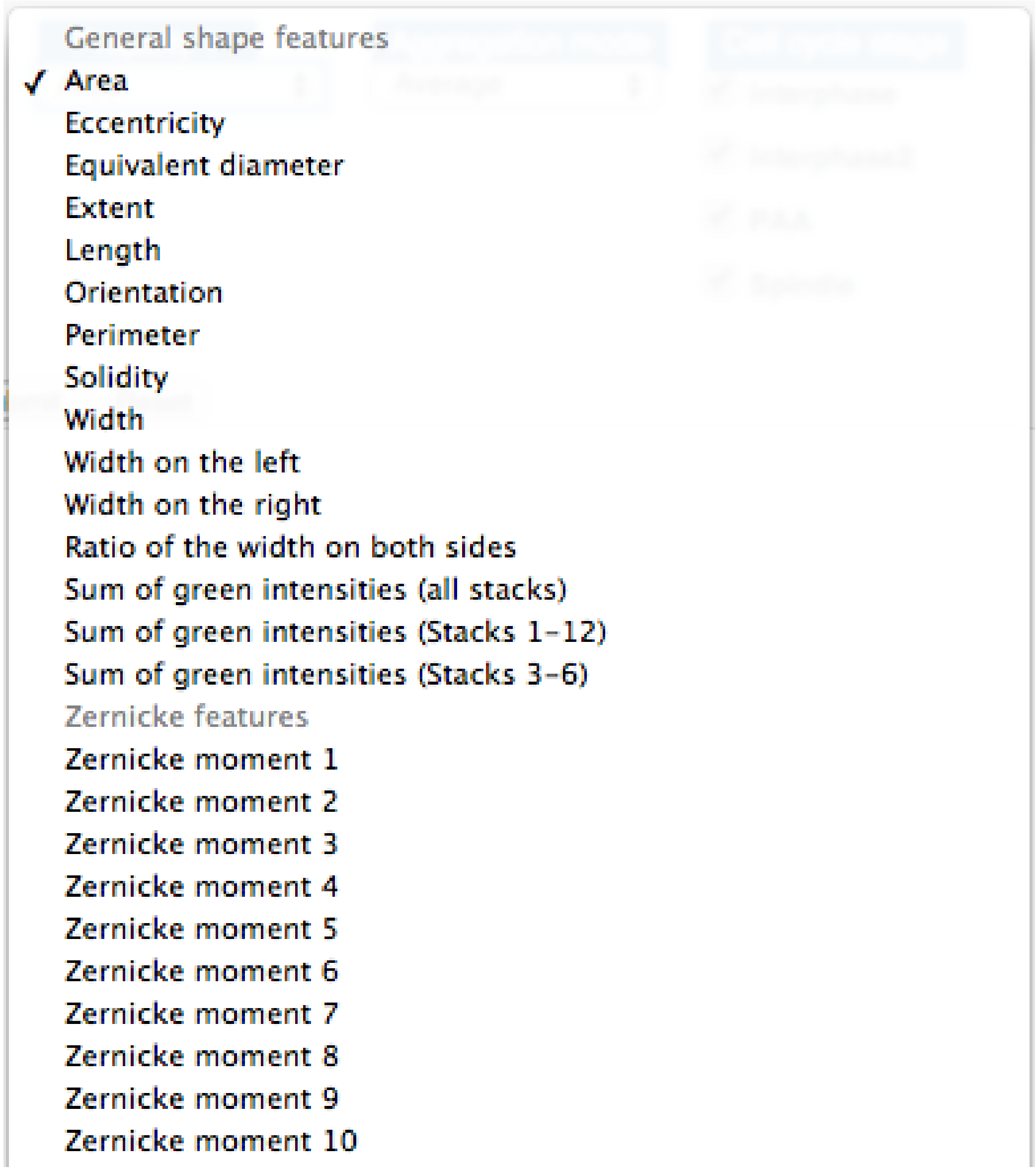

(Group level scatterplot only)The group level variables are aggregated from the descriptive level data. The aggregation mode selection panel allows the selection of how the group level value are supposed to be caluclated.

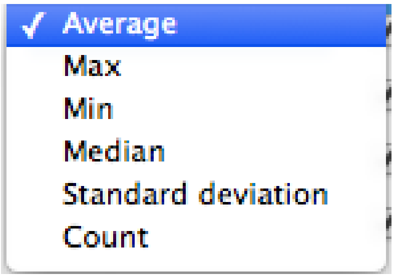

Finally, the descriptive data can be filtered via a filter property. That is, in this example only those cells are used in the query, which are in the selected cell cycle stage.

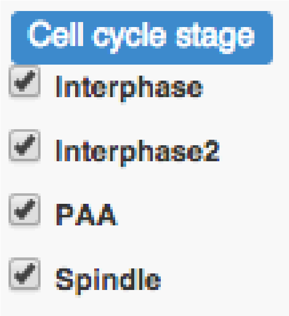

4.2 Filtering by annotation

The queried group objects can be filtered by their annotations. For example, only genes with a certain hit type associated are shown:

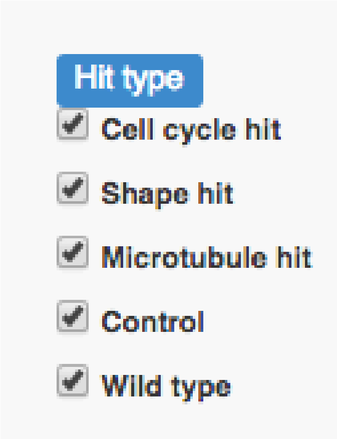

4.3 Group selection

Finally, we can select what group objects what we want to show on the plot. For descriptive level scatterplots, it is done by selection the object of interest from a list:

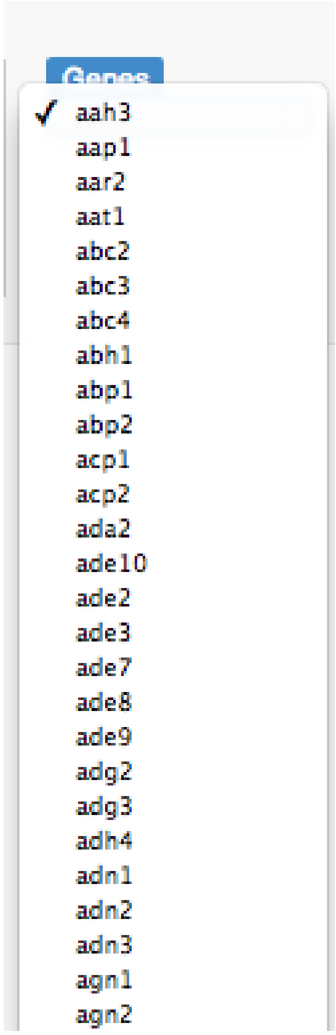

For group level scatterplots, the selection can be done by selecting objects from a selection menu:

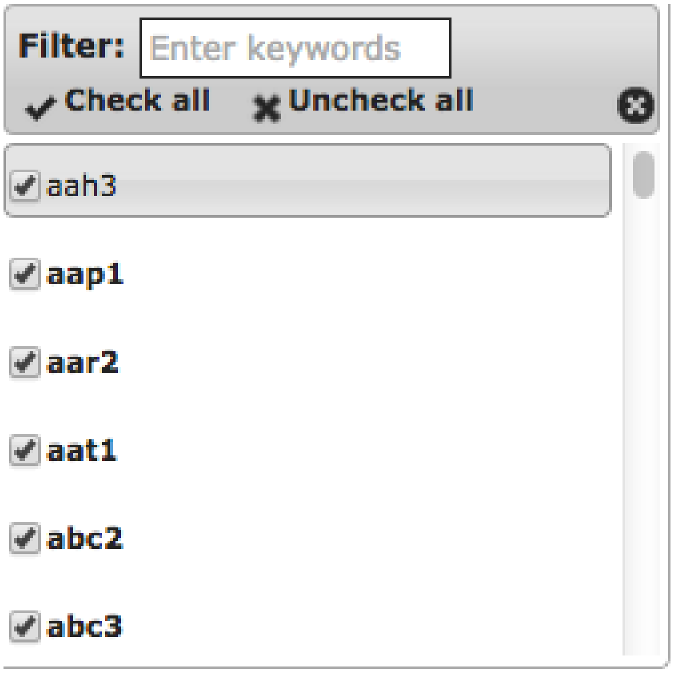

The search box on the top of the menu enables quick lookup of the objects included in the screen:

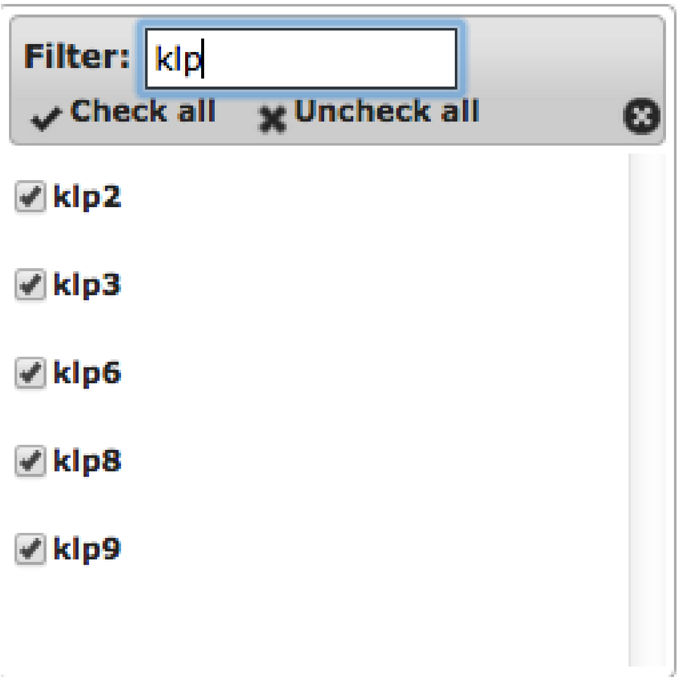

As an alternative, one could use the free text input by clicking the “Enter a list of genes” link below the selection box.

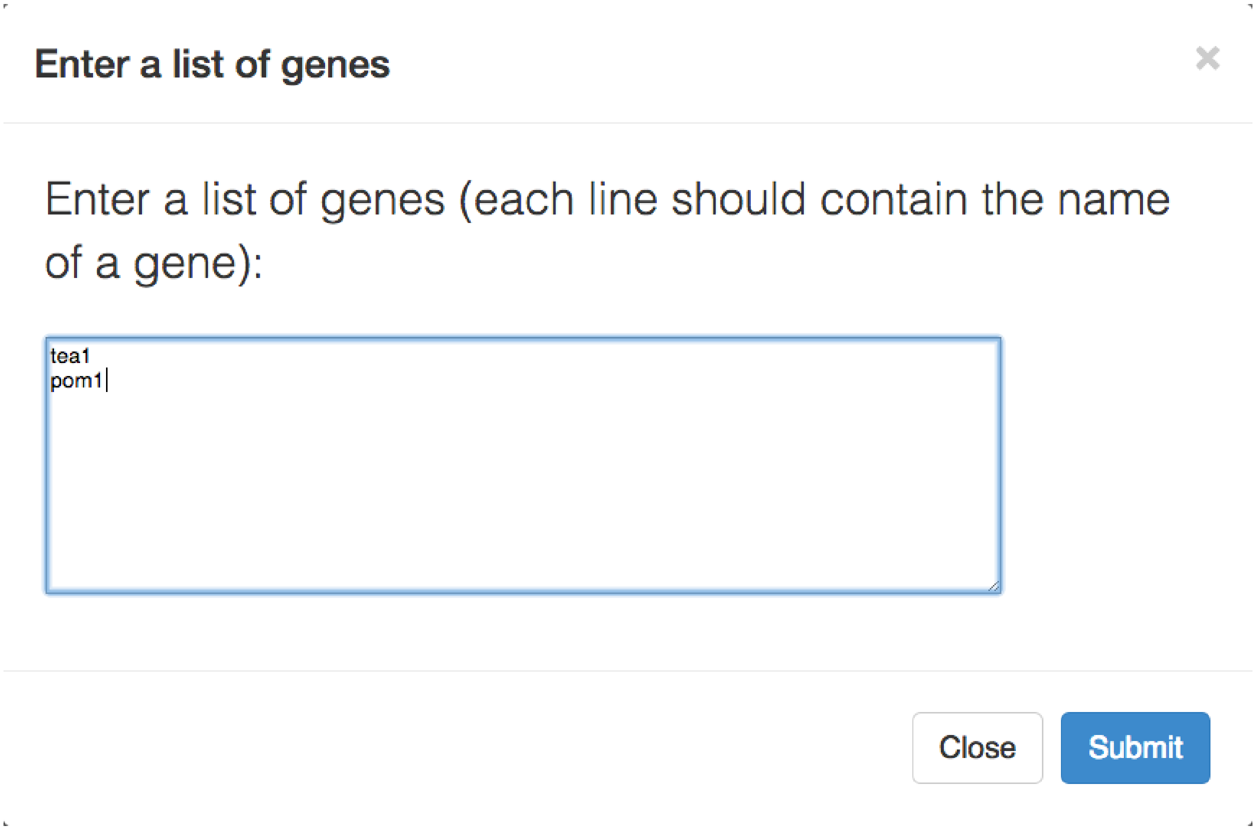

The entered gene names will be validated and the ones included in the screen will be selected. Once every option is selected, the submit button needs to be clicked. If you want to start over, click the Reset button which will turn every option to be their default settings.

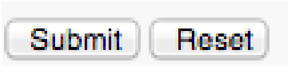

Scatterplots

A scatter plot shows two variables against each other in a 2D coordinate system. In a Mineotaur instance, there are two kinds of scatterplots: group level and descriptive level scatterplots. The query plot for a group level scatterplot looks like this:

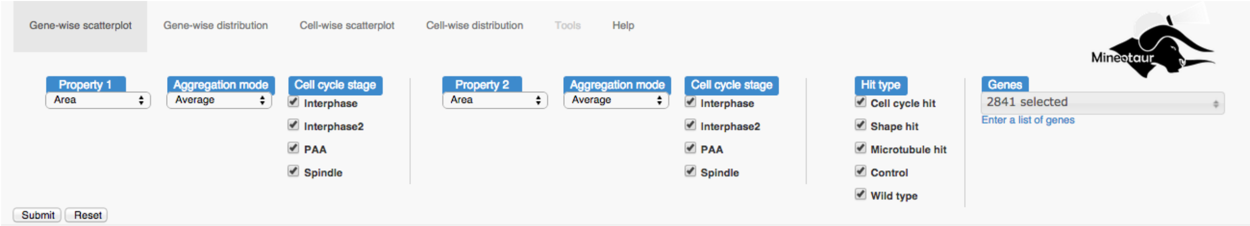

5.1 Using the scatterplot

Once you hit the submit button, the query is sent to the server and if there was data returned, a plot like this is displayed:

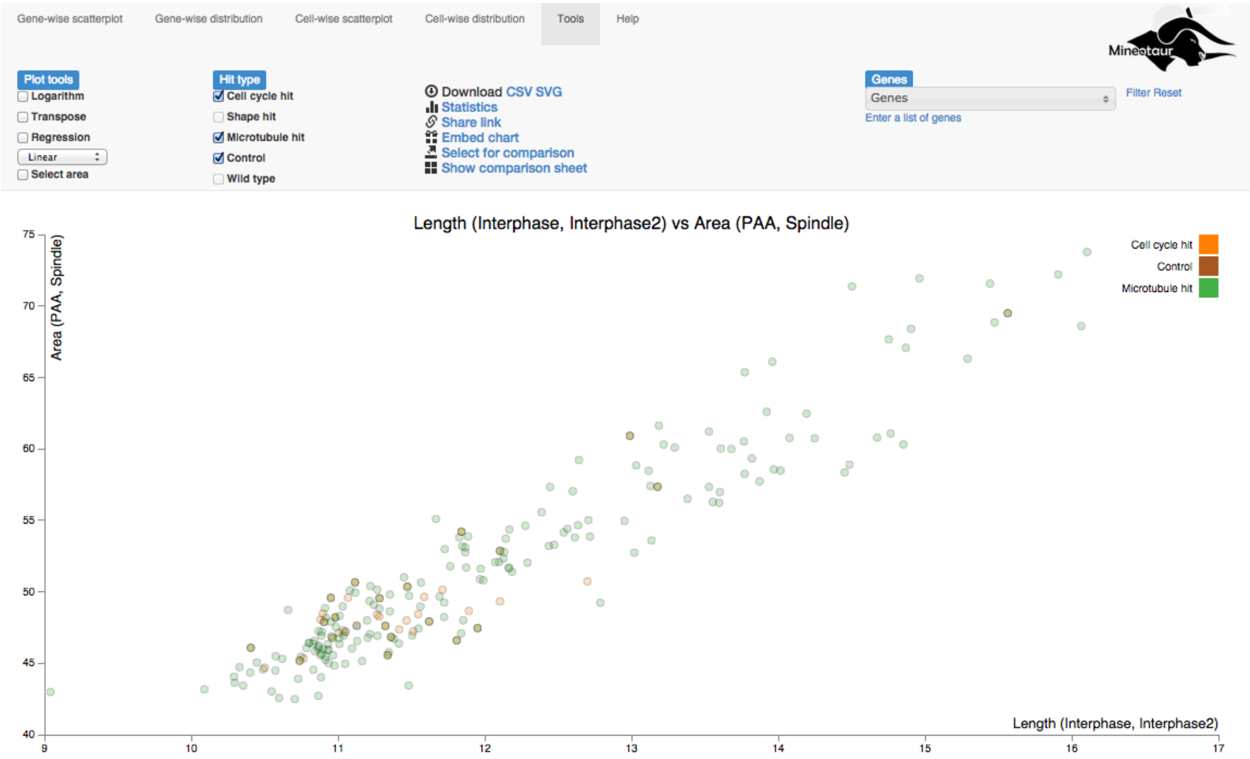

5.1.1 Coloring

The coloring of the data point are based on the colors associated to each annotation (hit) type, which can be seen in the top right corner of the plot:

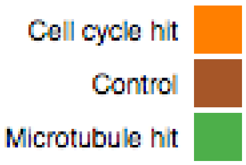

The nodes are also transparent, which enables the visual representation of multiple annotations (for which the coloring is the addition of the colors) as well as showing distribution of the data points.

5.1.2 Exploring invidiual data points

Name and values

To see the name of the underlying data point and the respective values for the queried variables, hover the mouse over the data point.

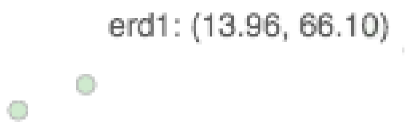

External resource

(Optional) Left clicking on the data points will open an external link associated to the object, e.g. the raw images used for analysis. This option only works if the external resource is provided during the instance generation.

Subqueries

By invoking the context menu (e.g. right-click in Windows or CMD+click in OSX) a subquery for the selected node can be created. That is, we can see the distribution of one of the queried variables or a descriptive scatterplot.

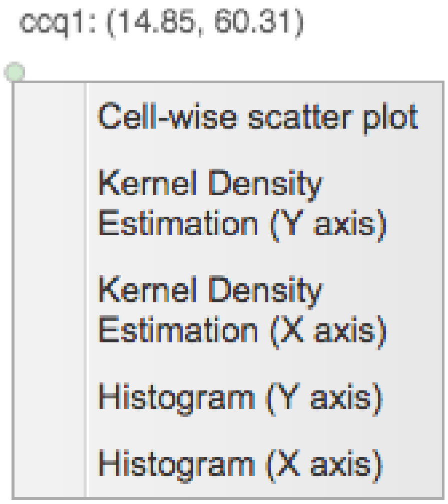

To go back to the original scatterplot, use the browser's back button.

5.2 Plot tools

Plot tools contain several to transform or analyze plots

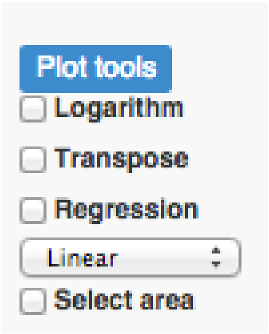

5.2.1 Logarithm

Clicking on the Logarithm checkbox transform the axes of the plot to logarithmic scale.

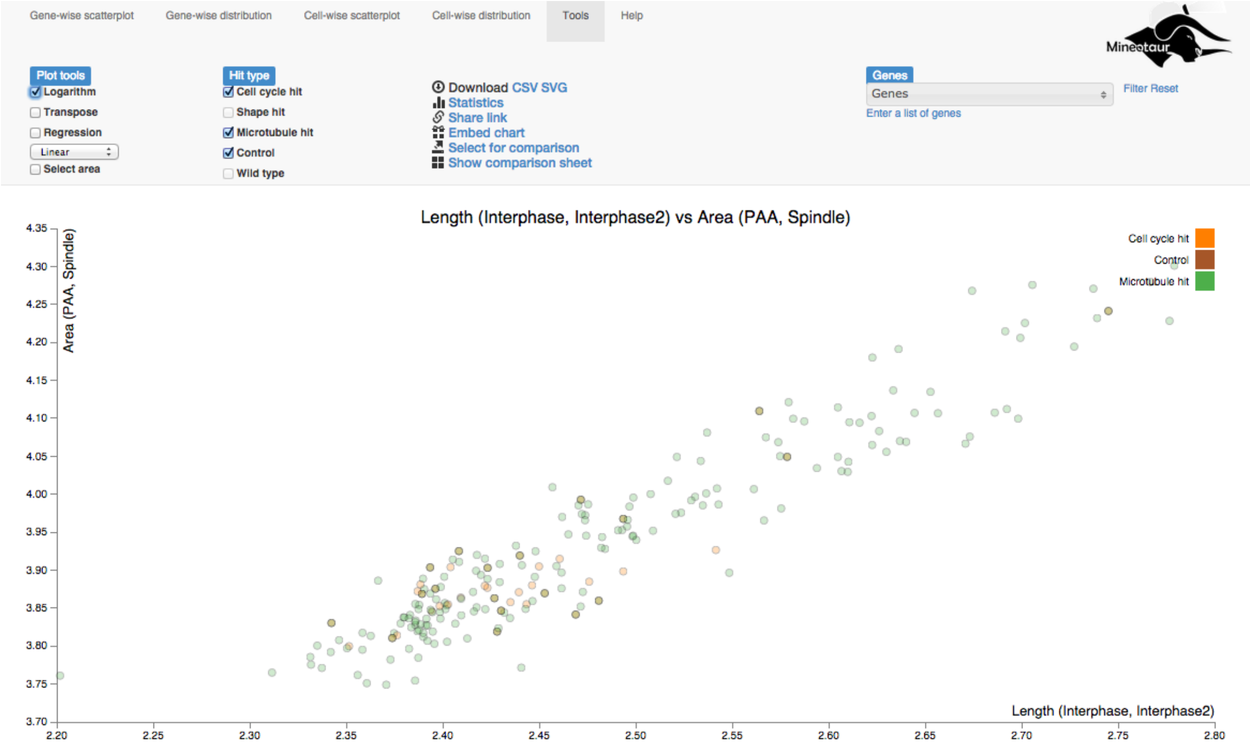

To go back to the original scale, untick the checkbox.

5.2.2 Transpose

Clicking on the Logarithm checkbox swaps the X-axis and the Y-axis.

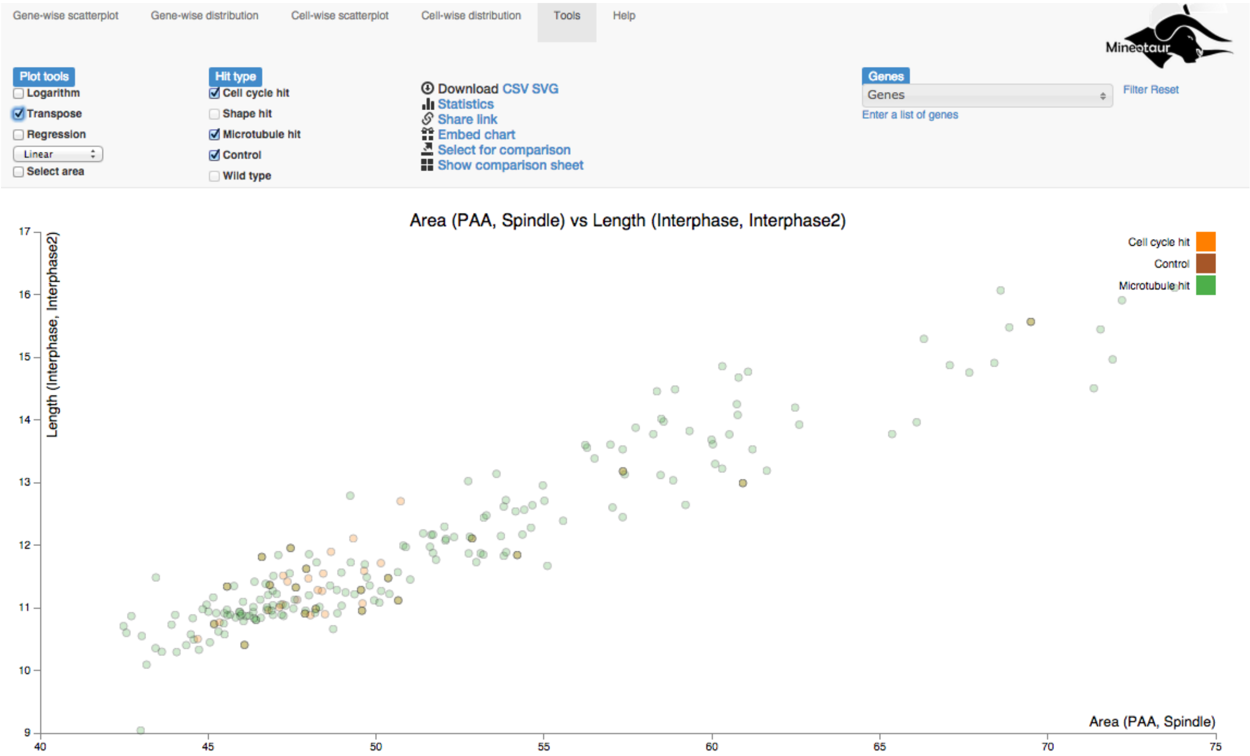

To go back to the original scale, untick the checkbox.

5.2.3 Regression

Clicking on the Regression checkbox fits a regression line on the data shown in the current plot. The type of the regression line can be selected from the selection box next to the checkbox.

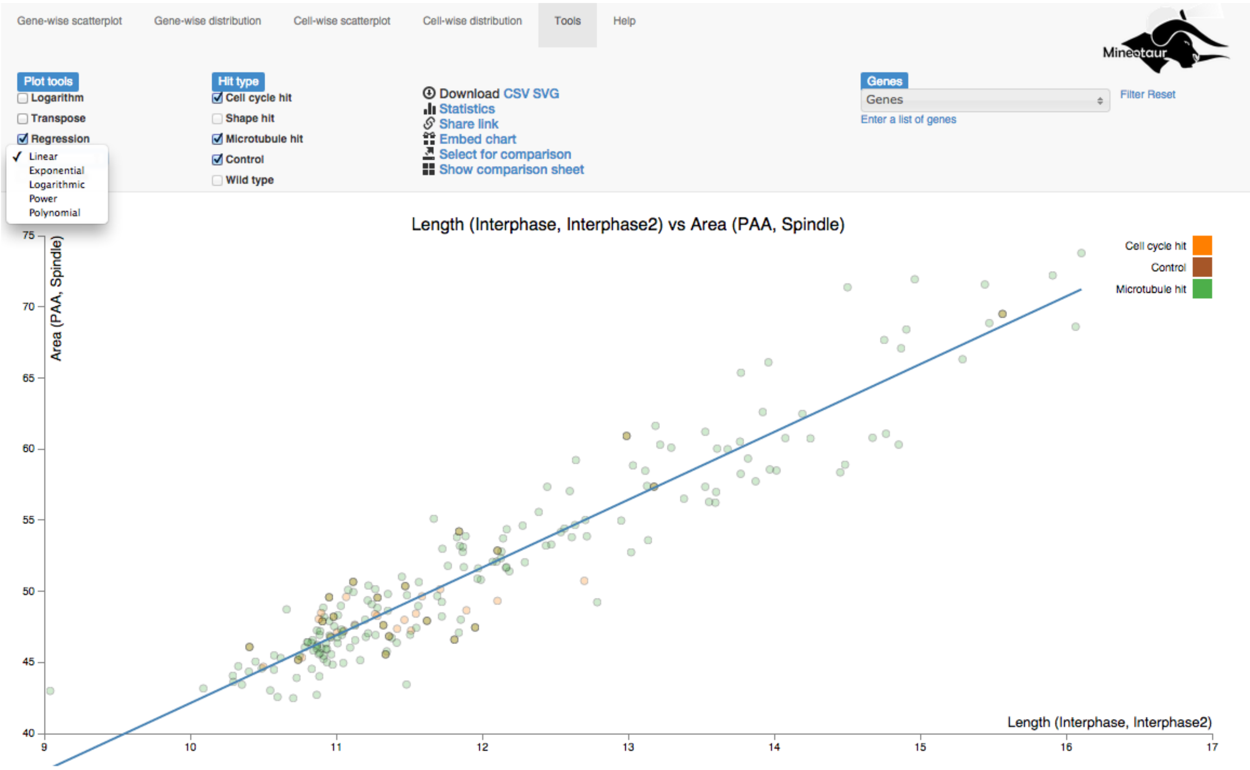

To see the correlation coefficient of the regression line, hover the mouse over the line:

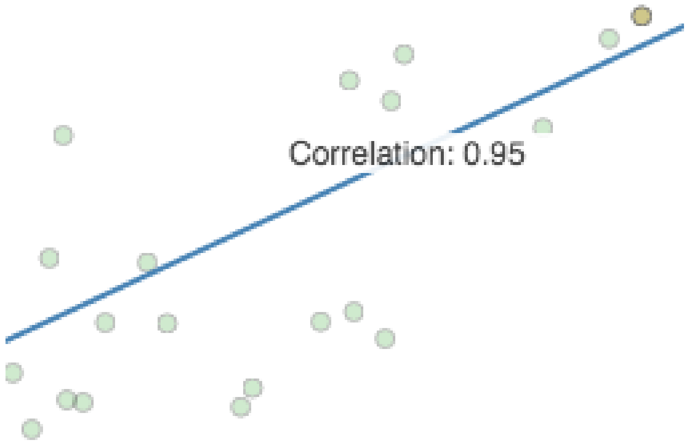

To remove the regression line, untick the checkbox.

5.2.4 Select area

To analyze a specific area of the plot, use the Select area tool. Checking the box transforms the cursor to an area selection tool, what you can use to draw a rectangle around the area to be selected:

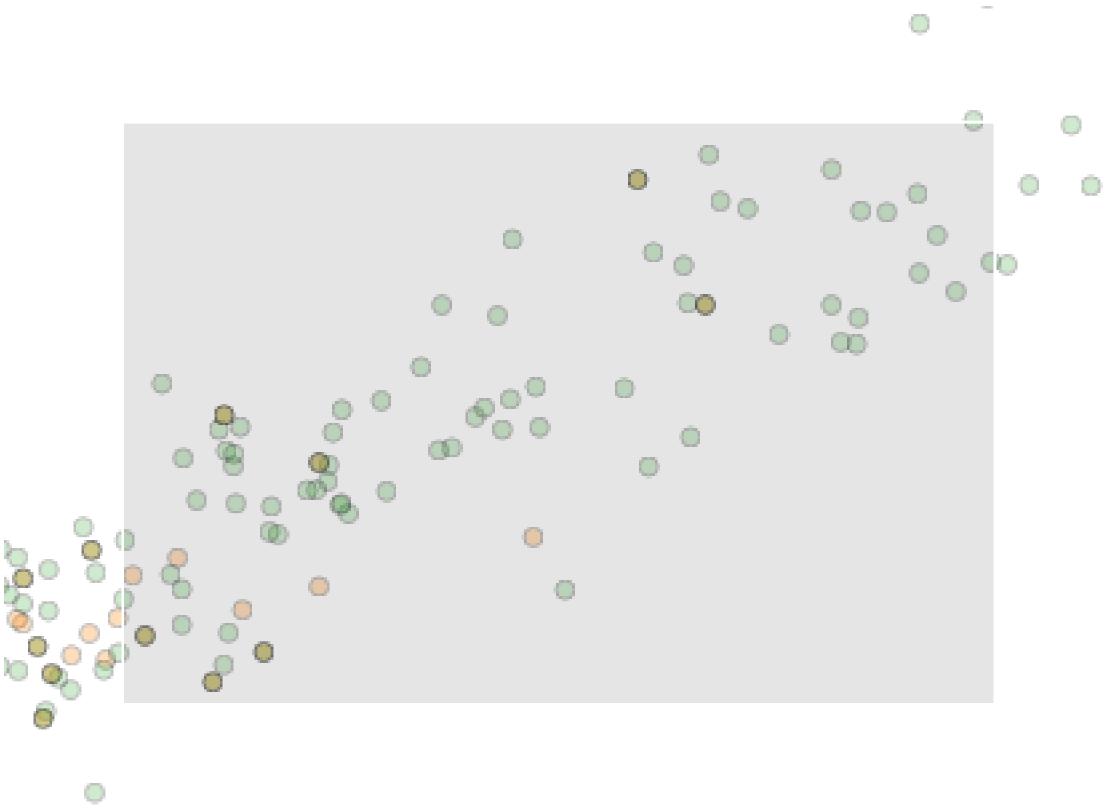

If you are satisfied with the selection, hover over the are and click on the Analyze button:

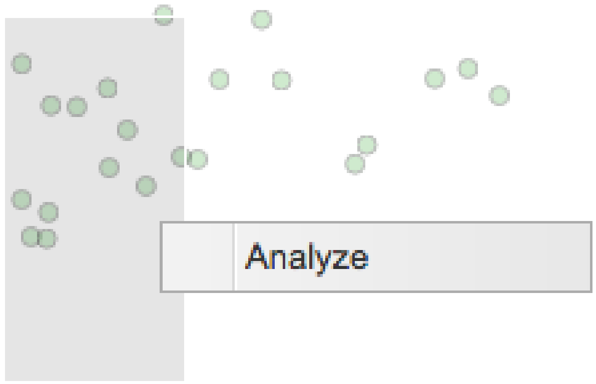

Then, a plot showing the data points from the selected are is shown. To go back to the previous plot, use the browsers Back button.

5.3 Visual filtering of nodes

Since scatterplots can be overcrowded, it is might be hardd to find individual objects on a plot. For example, genes of interest can be highlighted on a plot by selecting them from the provided list and clicking on the Filter link.

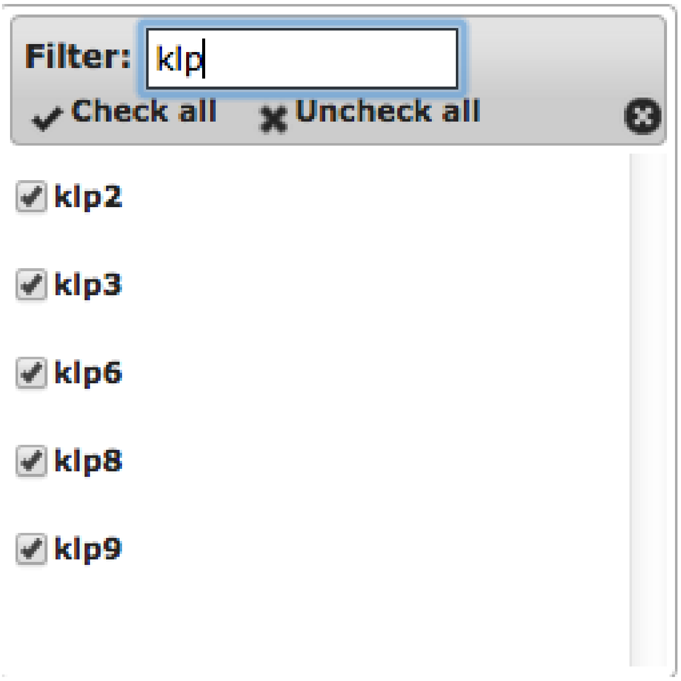

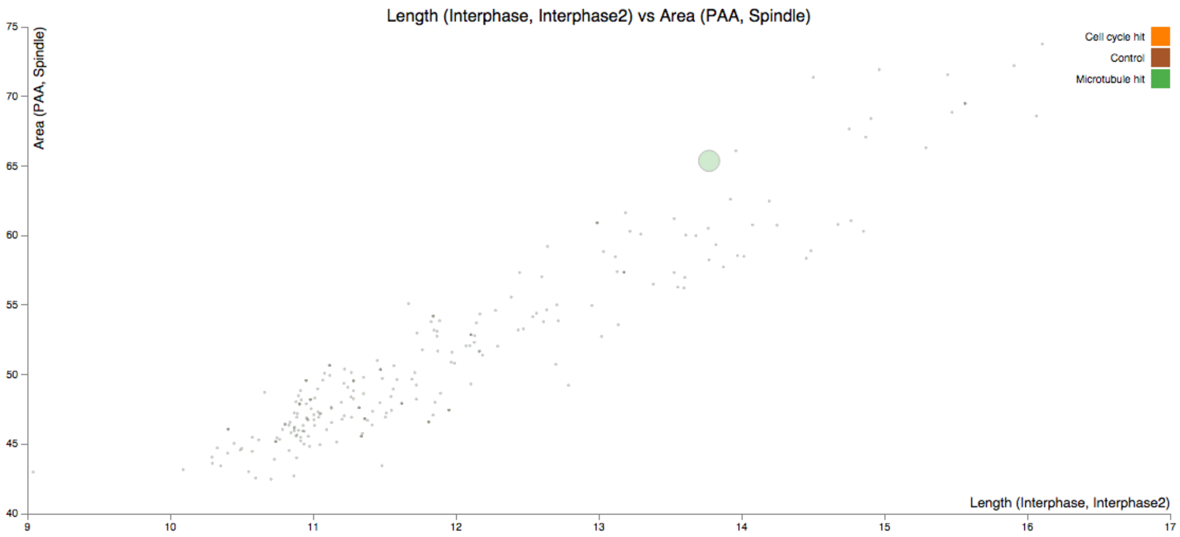

The highlighting can be reset by using the Reset link.

Distribution plots

Distribution plots provide a graphical representation of the distribution of a variable. In Mineotaur, there are three distribution plot types, which can be selected from the Plot type selection box from the Distribution plot query menu.

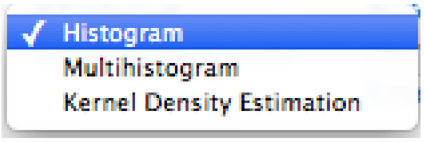

6.1 Histogram

Histograms shows the frequncy of variable values along the selected dataset. The binning of the histogram is automatically calculated based on the data.

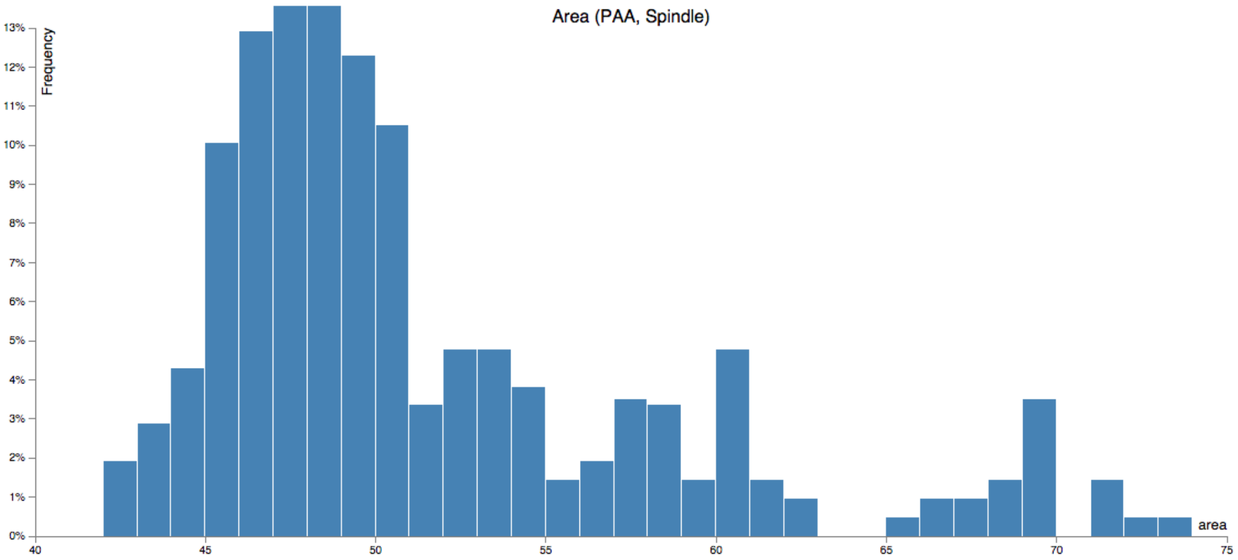

6.2 Multihistogram

(Group level only) Multihistograms shows the frequncy of variable values along the selected dataset where the data is split according to the annotations assigned to the data points. The histograms belonging to the annotations are shown

in different color. The legend is provided in the top right corner.

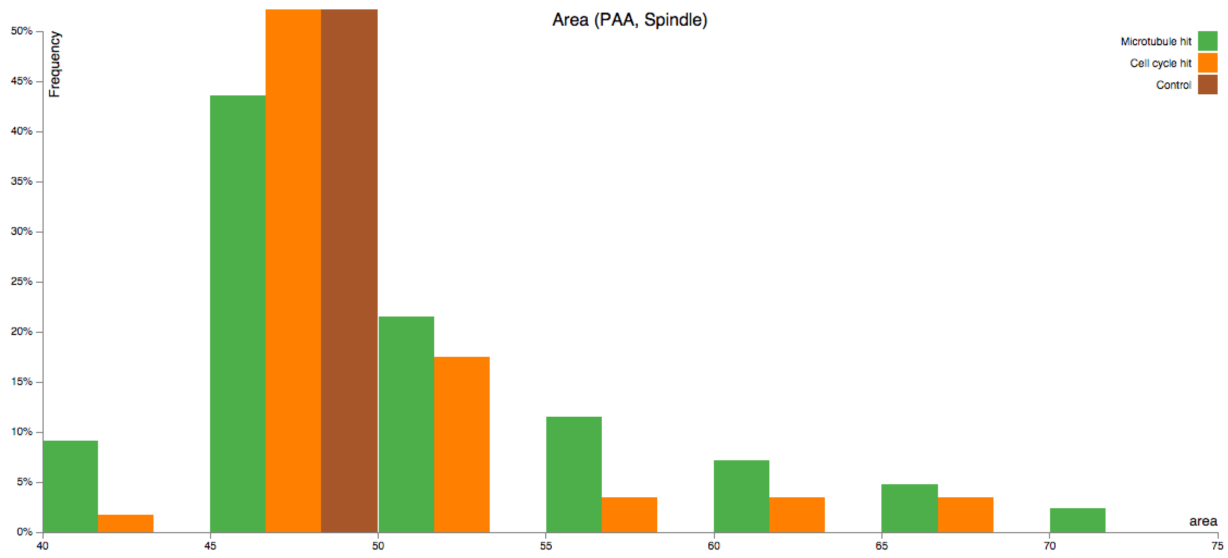

6.3 Kernel Density Estimation

Kernel Density Estimation plots show a continious approximation of the distribution with a Gaussian function fitted to the data. In group level plots, the different colors refer to the data point annotations. The legend is provided in the top right corner.

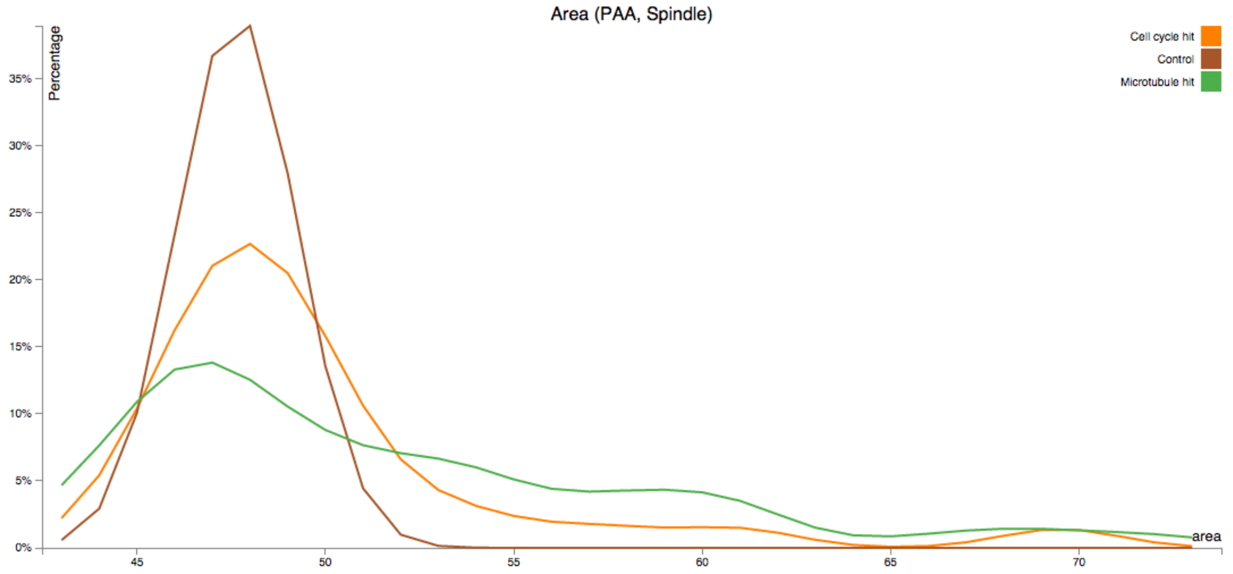

The Tools menu

Now, the Tools menu is enabled, allowing different action to be taken regarding the current plot.

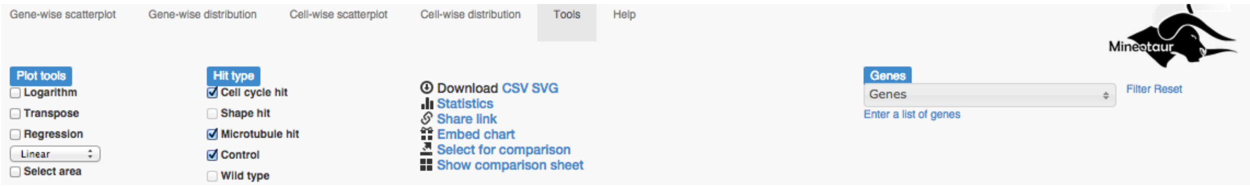

7.1 Filtering by hit Type

Just like for querying, the data points can be also filtered once the plot has been loaded. Unchecking and checking the boxes removes and puts back all queried data points for the respective hit types.

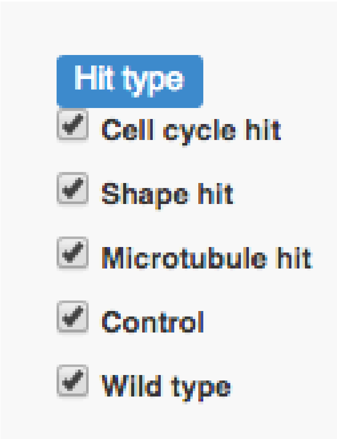

7.2 Other plot tools

There are also several tools to export, share or analyze the plots further.

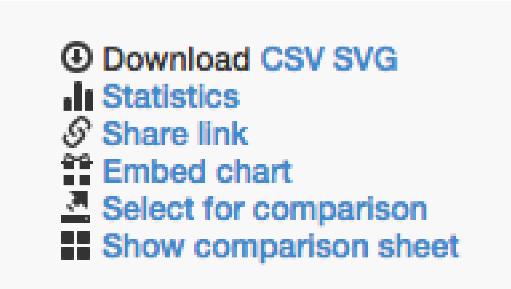

7.2.1 Download

By clicking on the CSV or SVG link, the raw data for creating the plot or the plot itself in a vectorgraphial format can be downloaded, respectively.

7.2.2 Statistics

The Statistic tool provide a summary on the underlying data of the plot.

Number of datapoints:
210

Property	Average	St. dev	Median	Min	Max
Length (Interphase, Interphase2)	11.96778991652581	1.358199280458194	11.509766828820538	9.040752042539683	16.10489999039735
Area (PAA, Spindle)	51.512042741556115	6.838754111393049	49.23927169669859	42.48120344827586	73.77593302222222

7.2.3 Share link

A permanent link for each plot can be generated with Mineotaur. The permanent link can be pasted in any browsers address bar and it will load the appropriate plot. The link has no expiry date, it invokes a mechanism which will recreate the plot.

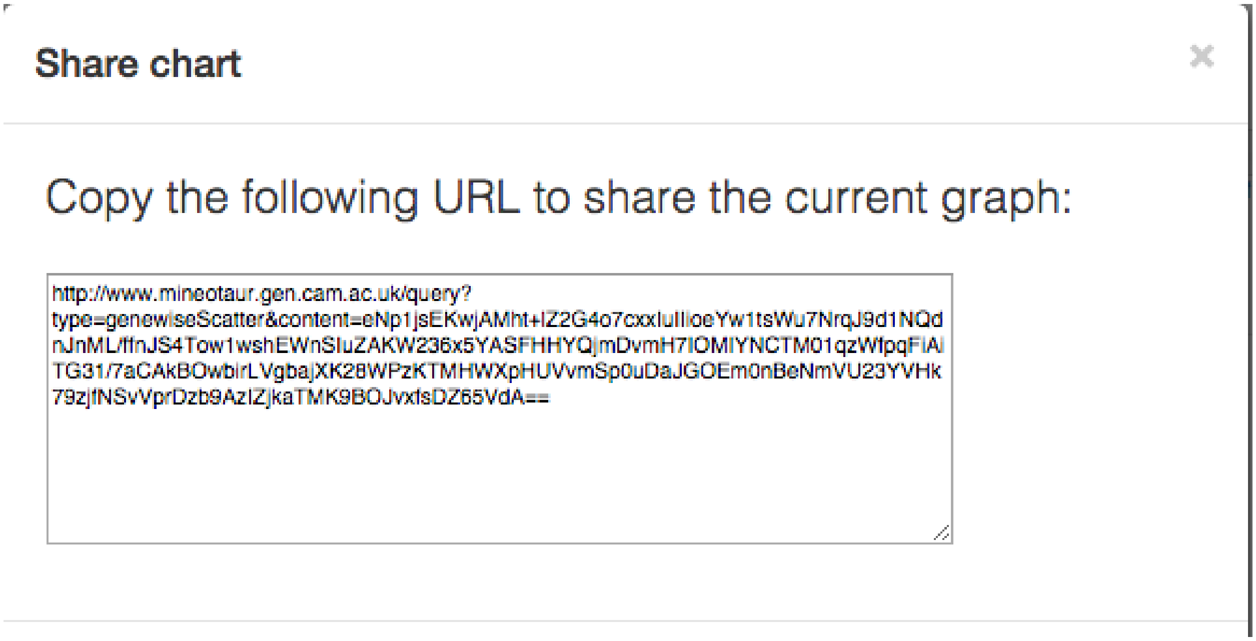

7.2.4 Embed link

Similarly to link sharing, an HTML snippet can be generated to each chart to embed it into an HTML page.

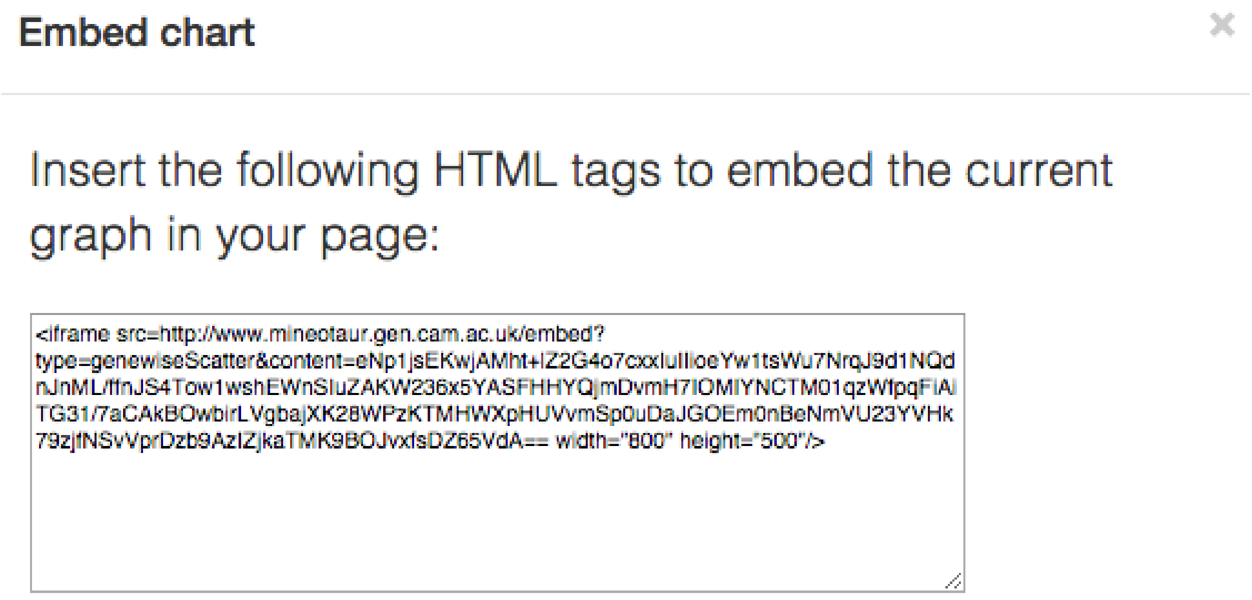

7.2.5 Comparing charts

Multiple (up to 4) plots can be shown alongisde each other on a comparison sheet. By clicking on the Select for comparison link, the current plot is copied to the next available position in the comparison sheet.

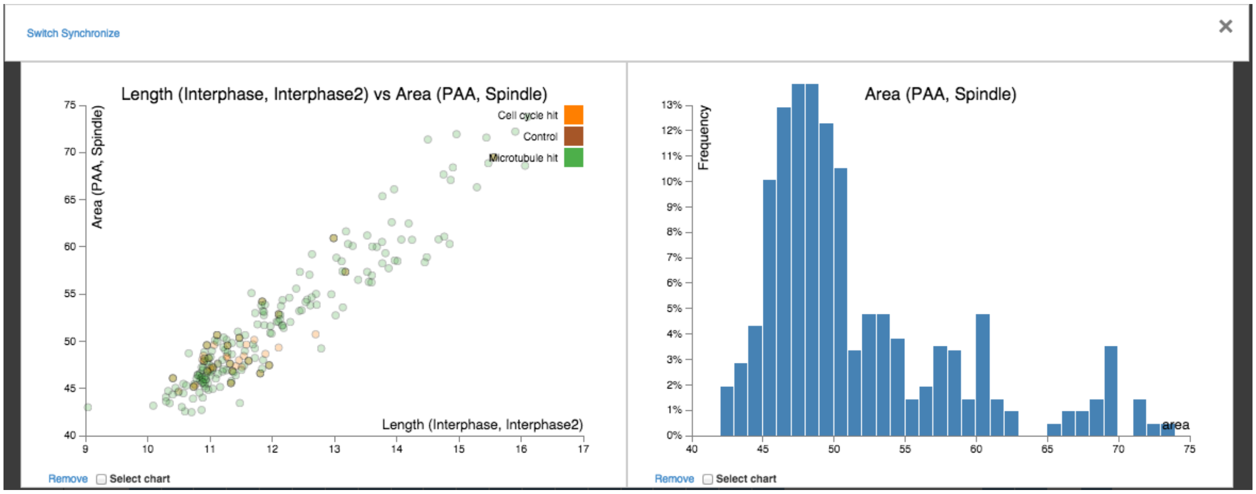

Once the plots are loaded, they can be removed, switched and plots from the same kind can be synchronized. That is, their axes are set to the same value, allowing straightforward visual comparison.

Developing Mineotaur

8.1 Software used to create Mineotaur

8.1.1 Server side:

- Programming language: Java 8
- Build system: Apache Maven
- Database: Neo4j
- Web framework: Spring Boot
- Template engine: Thymeleaf
- Test framework: TestNG
- Continous integration tool: Travis CI
- Bytecode manipulation tool: Javassist
- Command line parsing: Apache Commons CLI
- Math package: Apache Commons Math

8.1.2 Client side:

- Script and markup languages: Javascript, HTML 5, CSS
- Front-end framework: Twitter Bootstrap
- Visualization: D3
- HTML manipulation: jQuery
- jQuery UI library: jQuery-UI
- jQuery spinner: spin.js
- jQuery blockUI plugin: jquery.blockui.js
- jQuery form plugin: jquery.form.js
- jQuery history plugin: history.js
- jQuery context menu: jeegoocontext
- jQuery modal widget: Magnific Popup

- jQuery multiselect widget: jQuery UI MultiSelect widget
- jQuery modal widget: Magnific Popup
- AMD framework: RequireJS
- General utility collection: Underscore
- Math library: numbers.js
- Regression library: regression.js
- ZLib Javascript library: Pako

8.2 Architecture of Mineotaur

The Mineotaur web server can be accessed from both a web interface and programatically using REST. The web server handles the interaction with the graph database containing the HT/HCS data.

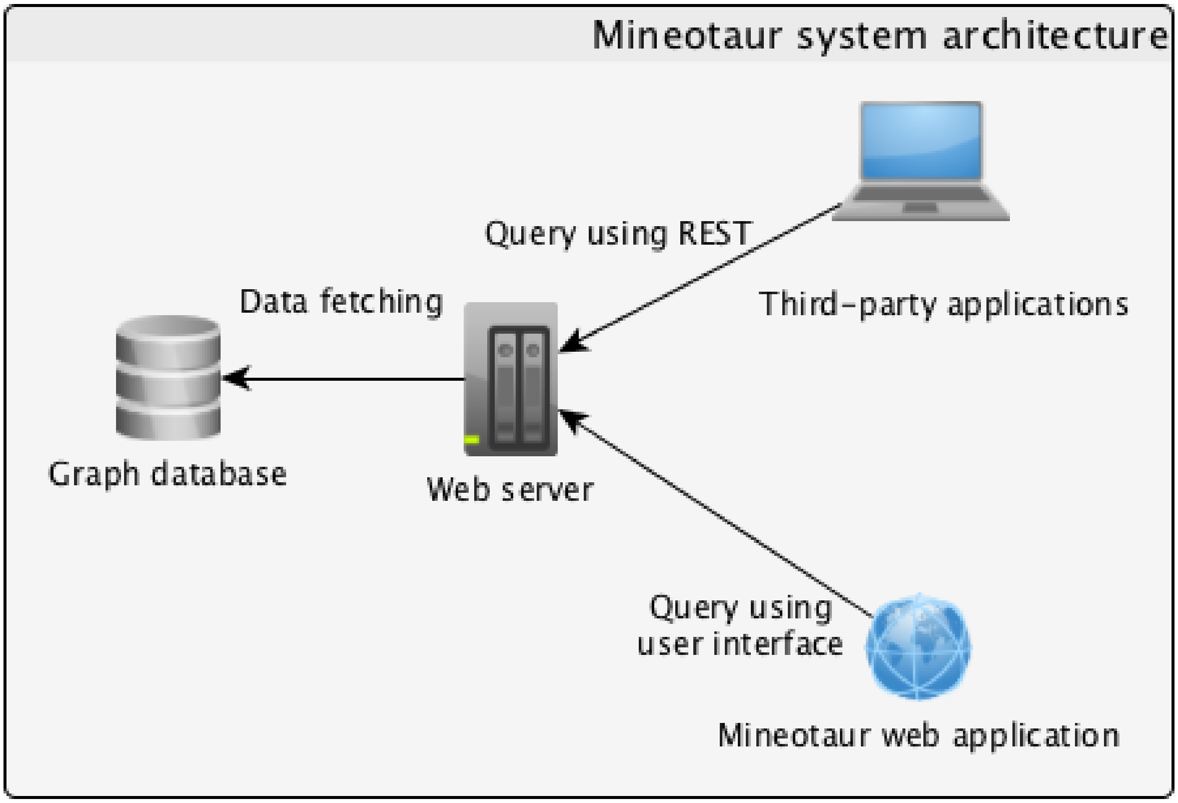

8.2.1 Server side architecture

The web server if based on the Spring Model-View-Controller (MVC), using Thymeleaf as a template engine. The data is stored in the Neo4j graph database. A web client can access the content by making an HTTP request to the server, which will query the appropriate data from the database and render a web page from a Thymeleaf template.

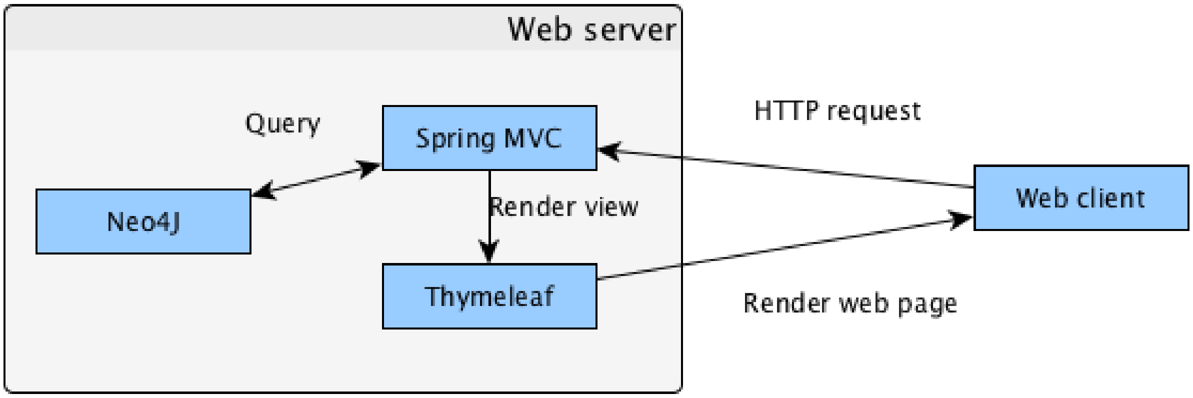

8.2.2 Client side architecture

On the client side, all interaction is done using a Javascript application. The application is modular, with different modules responsinble to handle events (Controller), carry data values (Context), manipulate web pages (UI), generate plots (Plot) and provide general functionalities (Utilities).

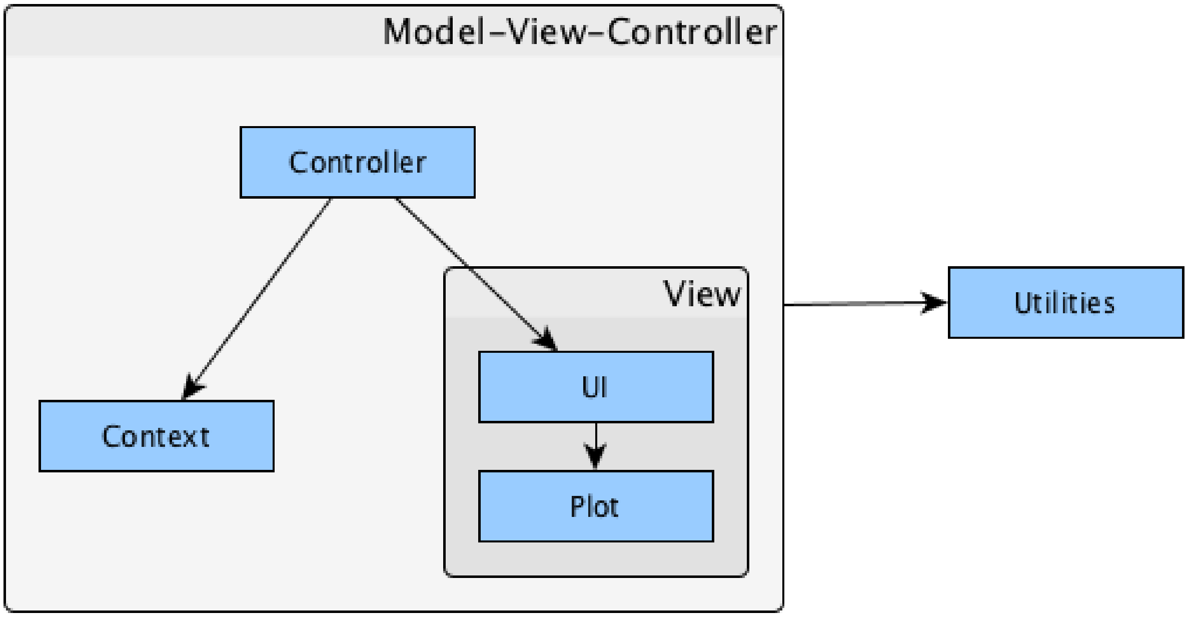

Features planned to be included in further releases of Mineotaur

1. Omero integration
2. REST client libraries
3. Time-lapse data handling
4. Network data handling

If you have any other suggestions, please let us now at info@mineotaur.org !

CHAPTER 10

Licence

Mineotaur: a visual analytics tool for high-throughput microscopy screens Copyright (C) 2014 Bálint Antal (University of Cambridge)

This program is free software: you can redistribute it and/or modify it under the terms of the GNU General Public License as published by the Free Software Foundation, either version 3 of the License, or (at your option) any later version.

This program is distributed in the hope that it will be useful, but WITHOUT ANY WARRANTY; without even the implied warranty of MERCHANTABILITY or FITNESS FOR A PARTICULAR PURPOSE. See the GNU General Public License for more details.

CHAPTER 11

Indices and tables

- genindex
- modindex
- search
